# Epidemiology, pathology and identification of *Colletotrichum* including a novel species associated with avocado (*Persea americana*) anthracnose in Israel

**DOI:** 10.1038/s41598-017-15946-w

**Published:** 2017-11-20

**Authors:** Gunjan Sharma, Marcel Maymon, Stanley Freeman

**Affiliations:** Department of Plant Pathology and Weed Research, Institute of Plant Protection, Agricultural Research Organization, The Volcani Center, Rishon LeZion, 7505101 Israel

## Abstract

Anthracnose disease caused by *Colletotrichum* species is a major constraint for the shelf-life and marketability of avocado fruits. To date, only *C*. *gloeosporioides sensu lato* and *C*. *aenigma* have been reported as pathogens affecting avocado in Israel. This study was conducted to identify and characterize *Colletotrichum* species associated with avocado anthracnose and to determine their survival on different host-structures in Israel. The pathogen survived and over-wintered mainly on fresh and dry leaves, as well as fresh twigs in the orchard. A collection of 538 *Colletotrichum* isolates used in this study was initially characterized based on morphology and banding patterns generated according to arbitrarily primed PCR to assess the genetic diversity of the fungal populations. Thereafter, based on multi-locus phylogenetic analyses involving combinations of ITS, *act*, *ApMat*, *cal*, *chs1*, *gapdh*, *gs*, *his3*, *tub*2 gene/markers; eight previously described species (*C*. *aenigma*, *C*. *alienum*, *C*. *fructicola*, *C*. *gloeosporioides sensu stricto*, *C*. *karstii*, *C*. *nupharicola*, *C*. *siamense*, *C*. *theobromicola*) and a novel species (*C*. *perseae*) were identified, as avocado anthracnose pathogens in Israel; and reconfirmed after pathogenicity assays. *Colletotrichum perseae* sp. nov. and teleomorph of *C*. *aenigma* are described along with comprehensive morphological descriptions and illustrations, for the first time in this study.

## Introduction

Avocado (*Persea americana* Mill.) is a high value crop grown in tropical and subtropical areas worldwide, including the Mediterranean basin. Israel is one of the prominent exporters of avocadoes in the world^1^, cultivated in approximately 7,000 hectares mainly along the Northern coastal plain to the Gaza strip in the South, and Eastern Galilee and Jordan Valley regions, yielding ca. 100,000 tons of fruit annually. Approximately 70% of the total production is exported to European countries while ca. 30% is utilized for local consumption^2^. Under subtropical Mediterranean conditions such as those prevailing in Israel, avocado fruit that set during the winter are seriously affected by post-harvest anthracnose fruit decay caused by *Colletotrichum* species^3^. Therefore, shelf life and marketability of avocado fruits are significantly reduced^4^. *Colletotrichum* is amongst the top ten fungal pathogens^5^; causing anthracnose disease in many economically important crops worldwide^6–12^. Anthracnose is characterized by the appearance of sunken necrotic black lesions along with the formation of orange conidial mass^13,14^. Infections of avocado occur in the orchard whereby conidia of the pathogen germinate, form appressoria but remain quiescent until fruit ripening after harvest^15^. Epidemiology of avocado anthracnose has not been studied in Israel and thus the source of inoculum affecting fruit has yet to be determined. It is plausible to assume that the source of conidia quiescently affecting fruit during the wet winters may originate from leaves or branches (dry or fresh) that are dispersed to the fruitlets and fruits formed during this period^16^. In tropical regions, it has been reported that the fungus survives between fruiting cycles on dried avocado leaves and twigs, either in the plant canopy or on the ground^17^.

Due to the worldwide importance of *Colletotrichum* as a plant pathogenic genus, accurate diagnosis is essential to improve bio-security and disease management strategies^6–8,18^. In particular, species belonging to the *C*. *gloeosporioides* species complex are pervasive, and are known to be common pathogens in tropical and subtropical areas^19–35^. Host-association and morphological characterization was previously used to identify *Colletotrichum* species^6,7^; but due to the overlapping morphological characters, a polyphasic approach is now recommended for accurate species identification within this genus^18,36,37^. Following the major taxonomic re-assessments in *Colletotrichum* ^9–12,38–41^, it is crucial to update the existing host-pathogen records. The recommended genes to be used in the phylogenetic analysis of the major *Colletotrichum* species complexes are respectively: Acutatum/Destructivum/Orbiculare/Spaethianum/Truncatum species complexes – *act* (actin), *chs1* (chitin synthase), *gapdh* (glyceraldehyde-3-phosphate dehydrogenase), *his3* (histone), ITS (5.8 S ribosomal RNA and the flanking internal transcribed spacer regions), *tub2* (β-tubulin); Boninense species complex – *act*, *cal* (calmodulin), *chs1*, *gapdh*, *his3*, ITS, *tub2*; Dematium/Gigasporum/Graminicola species complexes – *act*, *chs1*, *gapdh*, *ITS*, *tub2*; and the Gloeosporioides species complex - *act*, *cal*, *chs1*, *gapdh*, ITS^9–12,38–41^. Within the *C*. *gloeosporioides* species complex, use of the intergenic region between *apn2* and *Mat1*-*2* genes (*ApMat*) has been shown to be effective in cryptic species delimitation^21,24,27,42^.

To date, only two *Colletotrichum* species: *C*. *gloeosporioides sensu lato* and *C*. *aenigma* have been reported to be associated with avocado in Israel^11,43^. Due to the serious damage to avocado fruit caused by anthracnose disease in Israel, this study was initiated to (i) determine the genetic diversity of *Colletotrichum* using arbitrarily primed PCR (ap-PCR), multi-locus DNA sequence data coupled with morphology and pathogenicity assays; and (ii) determine on which plant organs the pathogen survives and over-winters under field conditions, in order to understand epidemiology of disease to help contribute to management practices.

## Results

### Fungal isolates and host infections

**Figure 1:**
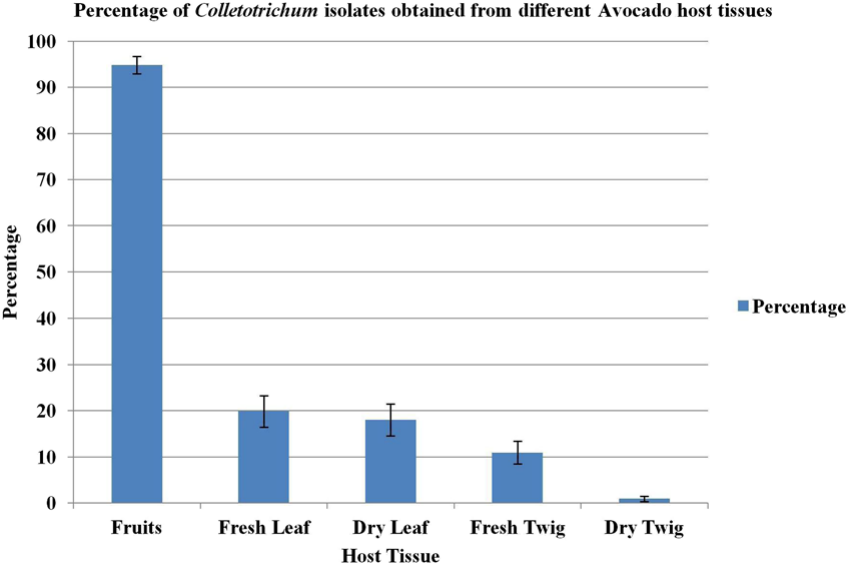
Bar diagram representing the percentage of *Colletotrichum* isolates obtained from different avocado host tissues. Bars represent the standard errors of mean for each tissue sample.

A total of 576 *Colletotrichum* isolates were recovered from different tissue (fruits, fresh leaves, fresh twigs, dry leaves and dry twigs) of avocado (Supplementary Table 1). Percentage of *Colletotrichum* isolates obtained from different host tissues is presented in Fig. 1. The *Colletotrichum* isolates were most readily isolated from infected fruits (94.88%), as compared to green leaves (19.87%), green twigs (10.93%) and dry leaves (18%) showing typical anthracnose symptoms. Low infection values of 0.9% were recorded for dry and dead twig tissues. Morphologically identical isolates recovered from the same tissue samples during isolation were discarded and 538 isolates were then selected for further molecular characterization.

### Assessment of genetic diversity

Amplification products were obtained for all the 538 *Colletotrichum* isolates from this study using four arbitrarily primed PCR (ap-PCR) primers: (CAG)_5_, (GACA)_4_, (AGG)_5_ and (GACAC)_3_. A high level of genetic diversity was observed, categorizing the isolates into eight distinct genetic groups (Supplementary Text). Genetic variability between the representative isolates from each group is presented in Supplementary Fig. 1. Thirty-three isolates were then selected from the eight genetically distinct groups based on their geographical location and isolation-tissue, for further multi-gene phylogenetic analyses, pathogenicity testing and morphological characterization.

### Identification of species complex using ITS gene region

Following assessment of genetic diversity, the ITS gene region of the 33 isolates was sequenced for their preliminary identification to the species complex level. Based on NCBI-BLAST search results of the ITS sequences, 31 isolates belonged to the *C*. *gloeosporioides* species complex and two isolates belonged to the *C*. *boninense* species complex. Further phylogenetic analyses were performed according to the described set of gene markers for the respective species complexes^9,11^. NCBI accession numbers for the sequences generated in this study appear in Tables 1 and 2.

**Table 1:** GenBank accession numbers of the *Colletotrichum* isolates belonging to the *C*. *gloeosporioides* species complex sequenced for the ITS, *gapdh*, *act*, *tub2*, *cal*, *ApMat* and *gs* gene sequences from this study (N.S. = not sequenced, ^T^ = type strain of the newly described species, in bold).

Isolate	Taxon name	ITS	*gapdh*	*act*	*tub2*	*cal*	*ApMat*	*gs*
GA050	*C*. *aenigma*	KX620303	KX620237	KX620140	KX620336	KX620201	N.S.	KX620270
GA098	*C*. *aenigma*	KX620307	KX620241	KX620144	KX620340	KX620205	KX620176	KX620274
GA223	*C*. *aenigma*	KX620314	KX620248	KX620151	KX620347	KX620212	N.S.	KX620280
GA230	*C*. *aenigma*	KX620316	KX620250	KX620153	KX620349	KX620215	KX620183	KX620282
GA415	*C*. *aenigma*	KX620327	KX620261	KX620164	KX620360	KX620226	N.S.	KX620293
GA512	*C*. *aenigma*	KX620331	KX620265	KX620168	KX620364	KX620230	KX620196	KX620296
GA524	*C*. *alienum*	KX620332	KX620266	KX620169	KX620365	KX620231	KX620197	KX620297
GA186	*C*. *fructicola*	KX620312	KX620246	KX620149	KX620345	KX620210	KX620181	KX620279
GA070	*C*. *gloeosporioides*	KX620304	KX620238	KX620141	KX620337	KX620202	KX620173	KX620271
GA077	*C*. *gloeosporioides*	KX620305	KX620239	KX620142	KX620338	KX620203	KX620174	KX620272
GA078	*C*. *gloeosporioides*	KX620306	KX620240	KX620143	KX620339	KX620204	KX620175	KX620273
GA125	*C*. *gloeosporioides*	KX620309	KX620243	KX620146	KX620342	KX620207	KX620178	KX620276
GA253	*C*. *nupharicola*	KX620319	KX620253	KX620156	KX620352	KX620218	KX620186	KX620285
GA039	*C*. *perseae* sp. nov.	KX620302	KX620236	KX620139	KX620335	KX620200	KX620172	KX620269
**GA100** ^**T**^ ** = CBS 141365 = HUJIHERB-902850-FUNGI**	*C*. *perseae* sp. nov.	KX620308	KX620242	KX620145	KX620341	KX620206	KX620177	KX620275
GA177	*C*. *perseae* sp. nov.	KX620311	KX620245	KX620148	KX620344	KX620209	KX620180	KX620278
GA272 = CBS 141366	*C*. *perseae* sp. nov.	KX620321	KX620255	KX620158	KX620354	KX620220	KX620188	KX620287
GA319	*C*. *perseae* sp. nov.	KX620322	KX620256	KX620159	KX620355	KX620221	KX620189	KX620288
GA320	*C*. *perseae* sp. nov.	KX620323	KX620257	KX620160	KX620356	KX620222	KX620190	KX620289
GA335	*C*. *perseae* sp. nov.	KX620325	KX620259	KX620162	KX620358	KX620224	KX620192	KX620291
GA341	*C*. *perseae* sp. nov.	KX620326	KX620260	KX620163	KX620359	KX620225	KX620193	KX620292
GA424	*C*. *perseae* sp. nov.	KX620329	KX620263	KX620166	KX620362	KX620228	KX620194	KX620294
GA131 = CBS 141364	*C*. *siamense*	KX620310	KX620244	KX620147	KX620343	KX620208	KX620179	KX620277
GA228	*C*. *siamense*	KX620315	KX620249	KX620152	KX620348	KX620214	KX620182	KX620281
GA250	*C*. *siamense*	KX620317	KX620251	KX620154	KX620350	KX620216	KX620184	KX620283
GA252	*C*. *siamense*	KX620318	KX620252	KX620155	KX620351	KX620217	KX620185	KX620284
GA263	*C*. *siamense*	KX620320	KX620254	KX620157	KX620353	KX620219	KX620187	KX620286
GA331 = CBS 141363	*C*. *siamense*	KX620324	KX620258	KX620161	KX620357	KX620223	KX620191	KX620290
GA435	*C*. *siamense*	KX620330	KX620264	KX620167	KX620363	KX620229	KX620195	KX620295
GA002	*C*. *theobromicola*	KX620300	KX620234	KX620137	KX620333	KX620198	KX620170	KX620267
GA006	*C*. *theobromicola*	KX620301	KX620235	KX620138	KX620334	KX620199	KX620171	KX620268

**Table 2:** GenBank accession numbers of the *Colletotrichum* isolates belonging to the *C*. *boninense* species complex sequenced for the ITS, *act*, *chs1*, *his3*, *tub2*, *cal* and *gapdh* gene sequences from this study.

Isolate	Taxon name	ITS	*act*	*chs1*	*his3*	*tub2*	*cal*	*gapdh*
GA206	*C*. *karstii*	KX620313	KX620150	KX620232	KX620298	KX620346	KX620211	KX620247
GA423	*C*. *karstii*	KX620328	KX620165	KX620233	KX620299	KX620361	KX620227	KX620262

### *ApMat* marker based phylogenetic analysis of the *C. gloeosporioides* species complex members

**Figure 2:**
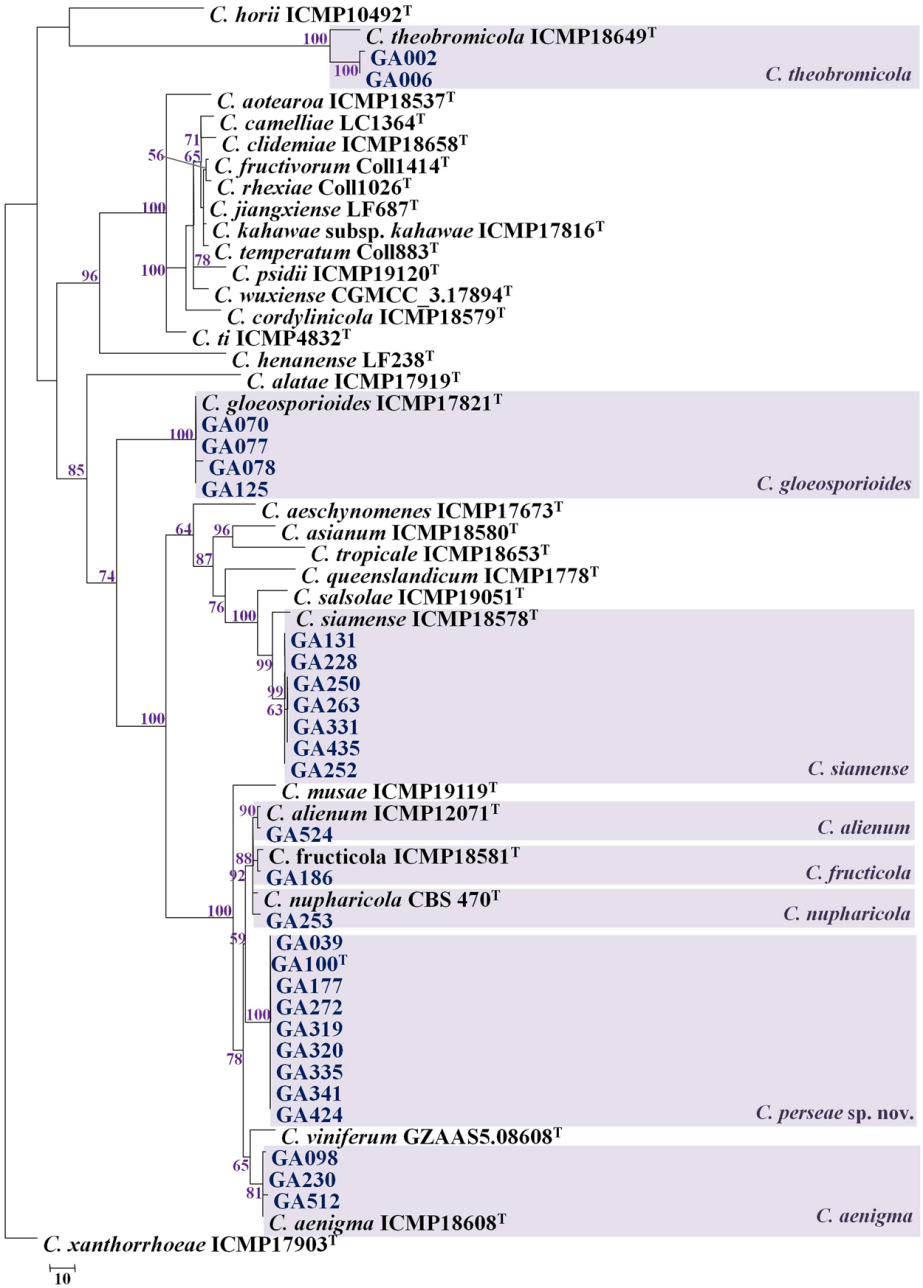
One of the two most parsimonious trees showing phylogenetic affinities of *Colletotrichum* isolates (highlighted in blue) belonging to the *C*. *gloeosporioides* species complex from Israel, obtained from heuristic search of the *ApMat* dataset. *Colletotrichum xanthorrhoeae* ICMP 17903 is used as an outgroup, and bootstrap support values exceeding 50%, are indicated at the nodes. (Type strains are marked with ^T^).

The *ApMat* dataset included 58 sequences and 944 characters including gaps. Forty-one characters from the ambiguously-aligned regions were excluded from the analysis. Of the remaining 903 characters, 398 characters were constant, 320 characters were parsimony-informative and 185 characters were parsimony-uninformative. MP analysis resulted in two trees and based on the KH test, the second tree was not significantly different as compared to the best tree (details not shown). One tree (TL = 832, CI = 0.773, RI = 0.932, RC = 0.721, HI = 0.227) generated in the MP analysis is shown in Fig. 2. The tree is rooted with *C*. *xanthorrhoeae* ICMP 17903. The recently described *Colletotrichum* species: *C*. *chengpingense*, *C*. *conoides*, *C*. *endophytica*, *C*. *grevilleae*, *C*. *grossum*, *C*. *hebeinse*, *C*. *helleniense*, *C*. *hystricis*, *C*. *liaoningense*, *C*. *proteae* and *C*. *syzygicola*; were not included in the analysis due to unavailability of *ApMat* sequences from the reference type strains. Bootstrap support values exceeding 50% for the observed branching pattern are shown next to the branch nodes. *ApMat* analysis resulted in strongly supported terminal clades (most branches having bootstrap support >70%). Using *ApMat* marker based phylogeny, 31 isolates belonging to the *C*. *gloeosporioides* species complex from this study were identified as *C*. *aenigma*, *C*. *alienum*, *C*. *fructicola*, *C*. *gloeosporioides sensu stricto*, *C*. *nupharicola*, *C*. *siamense*, *C*. *theobromicola* and one novel lineage described in this paper as *C*. *perseae* sp. nov.

*Colletotrichum* spp. isolates GA002 and GA006 clustered with the ex-type isolate of *C*. *theobromicola* (ICMP 18649) with 100% bootstrap support. *Colletotrichum* spp. isolates GA070, GA077, GA078 and GA125 clustered with the ex-type isolate of *C*. *gloeosporioides sensu stricto* (ICMP 17821) with 100% bootstrap support. *Colletotrichum* spp. isolates GA131, GA228, GA250, GA252, GA263, GA331 and GA435 clustered with the ex-type isolate of *C*. *siamense* (ICMP 18578) with 99% bootstrap support. *Colletotrichum* sp. isolate GA524 clustered with the ex-type isolate of *C*. *alienum* (ICMP 12071) with 90% bootstrap support. *Colletotrichum* sp. isolate GA186 clustered with the ex-type isolate of *C*. *fructicola* (ICMP 18581) with 88% bootstrap support. *Colletotrichum* sp. isolate GA253 clustered with the ex-type isolate of *C*. *nupharicola* (CBS 470) with 92% bootstrap support. *Colletotrichum* spp. isolates GA098, GA230 and GA512 clustered with the ex-type isolate of *C*. *aenigma* (ICMP 18608) with 81% bootstrap support. *Colletotrichum* spp. isolates GA039, GA100, GA177, GA272, GA319, GA320, GA335, GA341 and GA424 formed a distinct clade with 100% bootstrap support, adjacent to *C*. *fructicola*, *C*. *nupharicola* and *C*. *alienum*, described in this paper as *C*. *perseae* sp. nov.

### Multigene phylogenetic analyses of the *C. gloeosporioides* species complex members

Three *Colletotrichum* species *viz*. *C*. *fructivorum*, *C*. *rhexiae* and *C*. *temperatum* were excluded from the multi-gene analysis due to unavailability of reference sequences [*act*, *cal*, *gapdh*, glutamine synthase (*gs*)]. In addition, reference sequences were also lacking for *C*. *endophytica* (*tub2*); *C*. *chengpingense*, *C*. *hebeinse* (*cal*); and *C*. *chengpingense*, *C*. *conoides*, *C*. *endophytica*, *C*. *grossum*, *C*. *hebeinse*, *C*. *helleniense*, *C*. *hystricis*, *C*. *liaoningense* (*gs*). The 6-gene (*act*, *cal*, *gapdh*, *gs*, ITS, *tub2*) and 5-gene (*act*, *cal*, *gapdh*, ITS, *tub2*) phylogenetic analyses involved 69 taxa, including 31 isolates from this study and 38 reference isolates of the *C*. *gloeosporioides* species complex. *C*. *xanthorrhoeae* (ICMP 17903) was the designated outgroup. A further analysis including combined datasets of the *ApMat* marker and *gs* gene regions was also performed; to compare and validate the results of *ApMat* based phylogeny with the multigene phylogeny.

**Figure 3:**
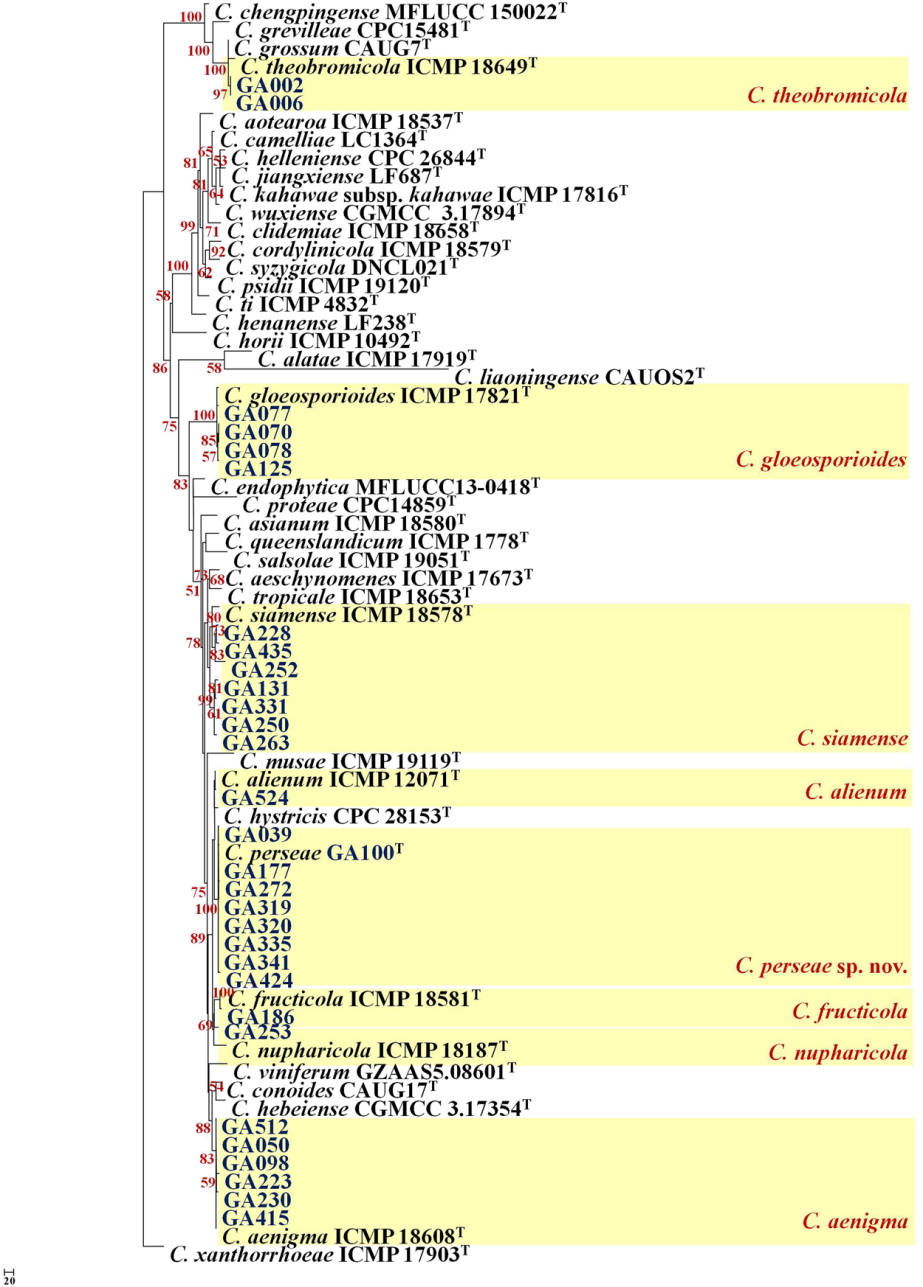
One of the 264 most parsimonious trees showing phylogenetic affinities of *Colletotrichum* isolates (highlighted in blue) belonging to the *C*. *gloeosporioides* species complex from Israel, obtained from heuristic search of the 6-gene (*act*, *cal*, *ITS*, *gapdh*, *gs*, *tub2*) dataset. *Colletotrichum xanthorrhoeae* ICMP 17903 is used as an outgroup, and bootstrap support values exceeding 50%, are indicated at the nodes. (Type strains are marked with ^T^).

The 6-gene dataset (*act*, *cal*, *gapdh*, *gs*, ITS, *tub2*) included 3628 characters with alignment gaps. Two hundred and twelve characters from the ambiguously aligned regions were excluded from the analysis. Of the remaining 3416 characters processed, 549 characters were parsimony-informative, 713 parsimony-uninformative and 2154 constant. Gene boundaries in the 6-gene dataset included; ITS: 1–590, *act*: 591–866, *cal*: 867–1651, *gapdh*: 1652–1955, *gs*: 1956–2878, *tub2*: 2879–3628). A heuristic search using PAUP resulted in 240 trees, of which one is shown in Fig. 3 (length = 2107, CI = 0.728, RI = 0.844, RC = 0.614, HI = 0.272). The topologies of the 240 trees were not significantly different (details not shown). The clustering pattern for the 31 isolates from 6-gene analysis was comparable to *ApMat*-based phylogeny and the overall bootstrap support for individual branches was typically strong [*C*. *aenigma* (88%), *C*. *fructicola* (100%), *C*. *gloeosporioides* (100%), *C*. *nupharicola* (69%), *C*. *perseae* sp. nov. (100%), *C*. *siamense* (78%), *C*. *theobromicola* (100%)]. The 5-gene (*act*, *cal*, *gapdh*, ITS, *tub2*) phylogenetic analysis (data not shown) was incapable of resolving *C*. *siamense*, *C*. *gloeosporioides*, *C*. *theobromicola*, *C*. *alienum*, *C*. *fructicola* and *C*. *nupharicola* into strongly supported clades. However, *C*. *aenigma* and *C*. *perseae* sp. nov. were resolved with 88% and 100% bootstrap support values, respectively.

The *ApMat*-*gs* dataset included 1867 characters including gaps (gene boundaries *ApMat*: 1–944, *gs*: 945–1867). The analysis involved 55 sequences, including 31 sequences from this study. Ninety-one characters from the ambiguously aligned regions were excluded from the analysis. Of the remaining 1776 characters, 965 were constant, 463 were parsimony-informative and 348 were parsimony-uninformative. The MP analysis resulted in one tree (TL = 1290, CI = 0.763, RI = 0.905, RC = 0.690, HI = 0.237) as shown in Supplementary Fig. 2. The bootstrap support values exceeding 50% for the observed branching pattern are indicated next to the branches. The *ApMat*-*gs* tree is strongly supported with high bootstrap values as compared to the 6-gene phylogeny [*C*. *aenigma* (100%), *C*. *alienum* (98%), *C*. *fructicola* (100%), *C*. *gloeosporioides* (100%), *C*. *nupharicola* (66%), *C*. *perseae* sp. nov. (100%), *C*. *siamense* (93%), *C*. *theobromicola* (100%)]. The topology of the *ApMat*-*gs* tree is in congruence with *ApMat*-phylogeny and the 6-gene phylogeny.

### 7-gene based phylogenetic analysis of the *C. boninense* species complex (*act*,* cal*,* chs1*, *gapdh*, *his3*, ITS, *tub2*)

**Figure 4:**
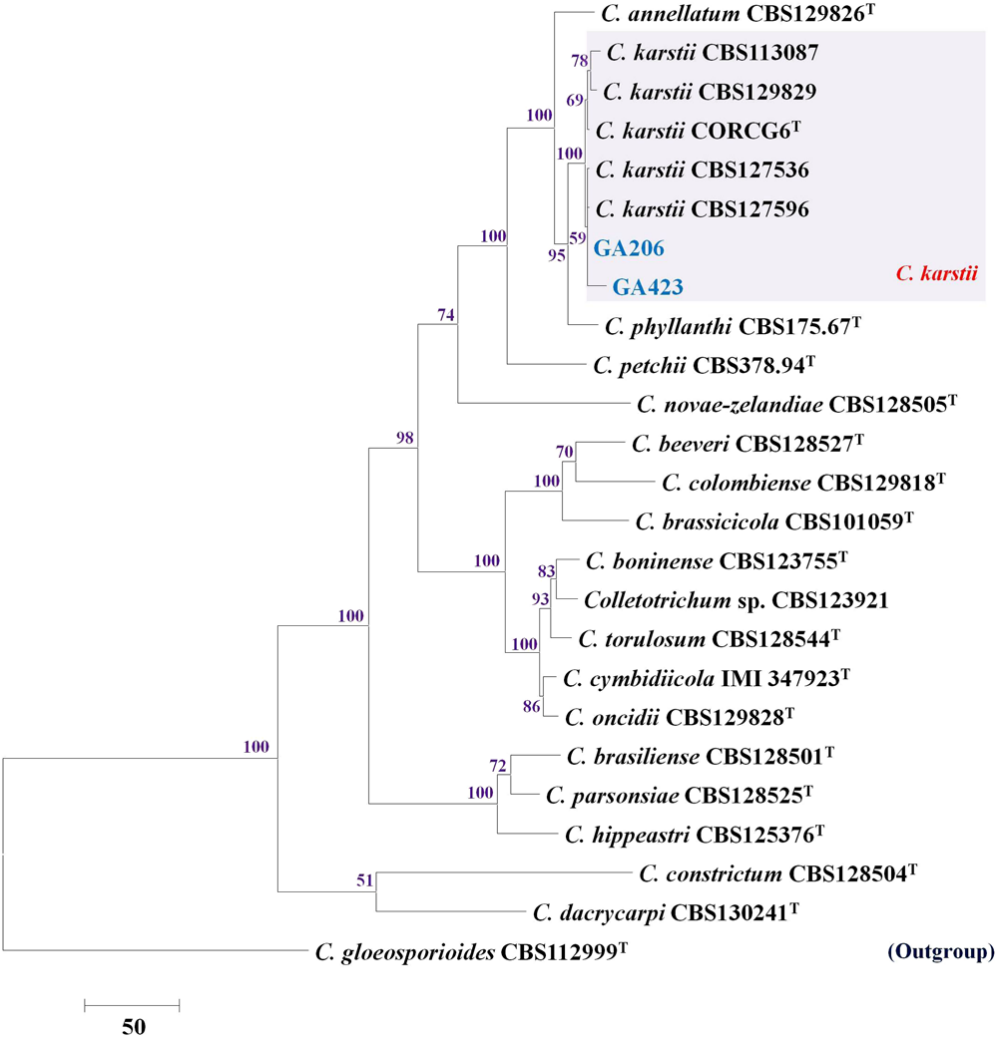
One of the most parsimonious trees showing phylogenetic affinities of *Colletotrichum* isolates (highlighted in blue) belonging to the *C*. *boninense* species complex from Israel, obtained from heuristic search of the 7-gene (*act*, *cal*, *chs1*, *ITS*, *gapdh*, *his3*, *tub2*) dataset. *Colletotrichum gloeosporioides* CBS 112999 is used as an outgroup, and bootstrap support values exceeding 50%, are indicated at the nodes. (Type strains are marked with ^T^).

The multi-gene analysis dataset included 2772 characters with alignment gaps (gene boundaries were, *chs1*: 1–280, ITS: 281–840, *act*: 841–1119, *cal*: 1120–1572, *gapdh*: 1573–1874, *his3*: 1875–2269, *tub2*: 2270–2772). The analysis involved 25 taxa, including 2 isolates from this study, 22 reference isolates belonging to the *C*. *boninense* species complex and the outgroup (*C*. *gloeosporioides* strain CBS 112999). Eighty-four characters from the ambiguously aligned regions were excluded from the analysis. Of the remaining 2688 characters, 1892 were constant, 396 were parsimony-informative and 400 were parsimony-uninformative. The MP analysis resulted in one tree (TL = 1341, CI = 0.759, RI = 0.800, RC = 0.607, HI = 0.241), as shown in Fig. 4. The two isolates from this study, GA206 and GA423, clustered within the *C*. *karstii* clade with 100% bootstrap support.

### Pathogenicity assay

**Figure 5:**
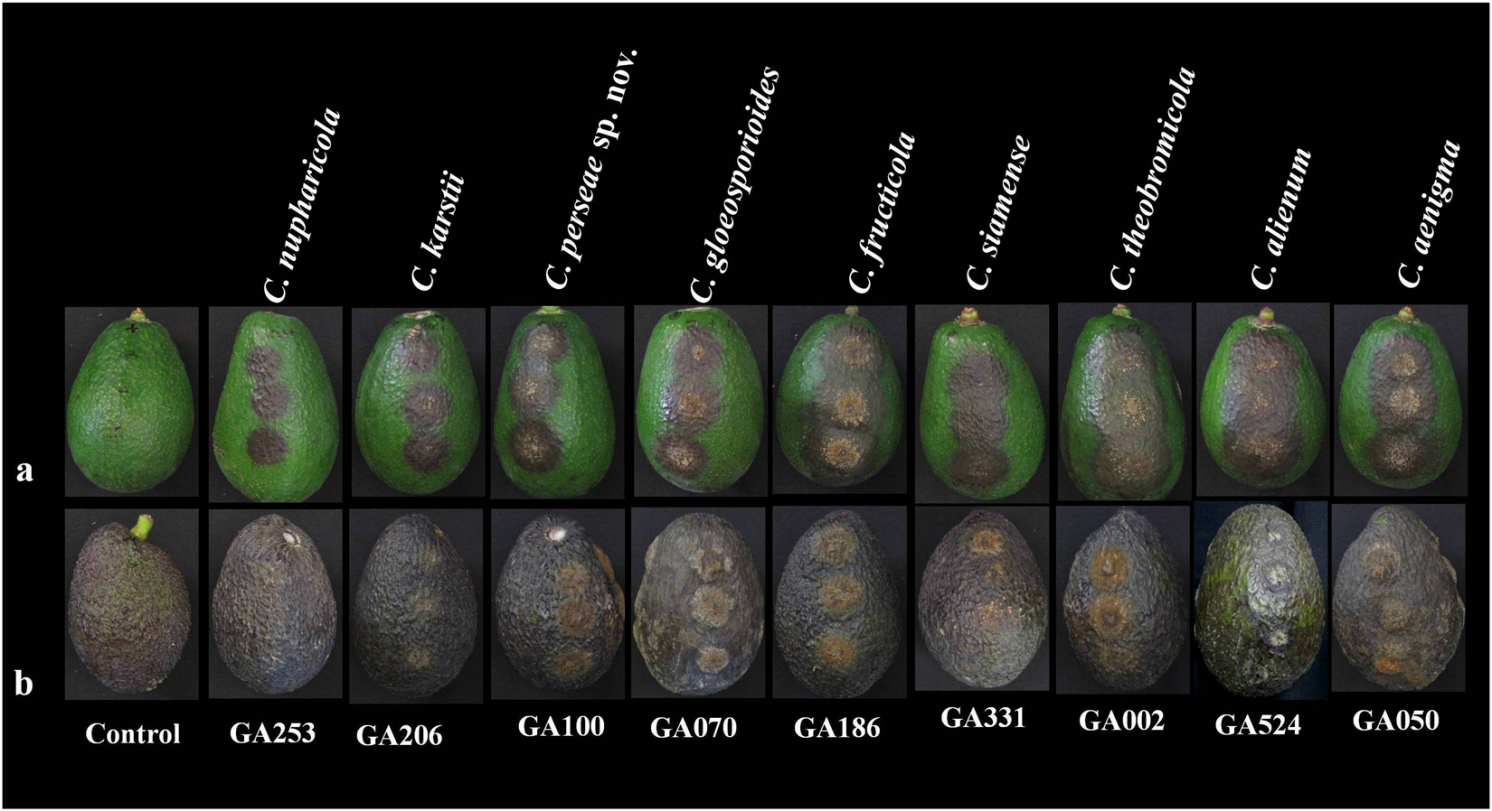
Results of pathogenicity assays of selected *Colletotrichum* species isolates (*C*. *aenigma* – GA050, *C*. *alienum* – GA524, *C*. *fructicola* – GA186, *C*. *gloeosporioides* – GA070, *C*. *karstii* – GA206, *C*. *nupharicola* – GA253, *C*. *perseae* sp. nov. – GA100, *C*. *siamense* – GA331, *C*. *theobromicola* – GA002) 7 days post inoculation on (**a**) Reed and (**b**) Hass avocado cultivars. Control fruit was mock-inoculated with sterile water.

The inoculated fruits developed typical anthracnose lesions around the wound (Fig. 5); however, disease development at the unwounded site was very limited or absent even after seven days post inoculation (data not shown). To validate Koch’s postulates, pathogens were re-isolated from the infected host tissues. The control fruits did not develop anthracnose symptoms. All *Colletotrichum* isolates from this study caused 100% disease incidence. *Colletotrichum aenigma* proved to be the most virulent pathogen of avocado in Israel with 92.6 ± 7.7% disease severity (Table 3). The next two virulent pathogens were *C*. *alienum* and *C*. *theobromicola* with 90.1 ± 6.7 and 88.9 ± 3.7% disease severity scores, respectively. The percent disease severity (PDS) scores for other isolates were: *C*. *siamense* (85.9 ± 4.3%), *C*. *fructicola* (85.2 ± 4.3%), *C*. *gloeosporioides* (82.7 ± 5.0%), *C*. *perseae* sp. nov. (80.2 ± 2.7%), *C*. *karstii* (67.9 ± 6.5%) and *C*. *nupharicola* (63.0 ± 14.7%). Pathogenicity assays were performed in triplicate, and similar results were obtained for each experiment (data not shown). Calculations for the results of PDS are provided in Table 3.

**Table 3:** Disease score (DS), percent disease incidence (PDI) and percent disease severity (PDS) for each fruit (cultivar), 7 days post inoculation with representative strains of *Colletotrichum* species.

Isolate	Disease Score (DS) on a 0–9 scale													**PDI** (%)	**Mean PDS** (%)
	Control fruit	Replicate 1 (*Reed*)				Replicate 2 (*Reed*)				Replicate 3 (*Hass*)					
		Test fruit 1	Test fruit 2	Test fruit 3	PDS (%)	Test fruit 4	Test fruit 5	Test fruit 6	PDS (%)	Test fruit 7	Test fruit 8	Test fruit 9	PDS (%)		
GA050 (*C*. *aenigma*)	0	9	9	9	100%	9	9	9	100%	7	7	7	77.77%	100%	92.59 ± 7.66
GA524 (*C*. *alienum*)	0	7	9	9	92.59%	9	9	9	100%	7	7	7	77.77%	100%	90.12 ± 6.74
GA186 (*C*. *fructicola*)	0	7	9	9	92.59%	7	7	7	77.77%	9	7	7	85.18%	100%	85.18 ± 4.33
GA070 (*C*. *gloeosporoides*)	0	7	9	9	92.59%	7	7	7	77.77%	7	7	7	77.77%	100%	82.71 ± 5.00
GA206 (*C*. *karstii*)	0	7	7	7	77.77%	7	5	7	70.37%	7	1	7	55.55%	100%	67.89 ± 6.48
GA253 (*C*. *nupharicola*)	0	7	7	7	77.77%	7	7	7	77.77%	5	1	3	33.33%	100%	62.95 ± 14.66
GA100 = CBS 141365 (*C*. *perseae* sp. nov.)	0	7	7	7	77.77%	9	7	7	85.18%	7	7	7	77.77%	100%	80.24 ± 2.66
GA331 (*C*. *siamense*)	0	9	7	9	92.59%	7	7	7	77.77%	7	7	9	85.18%	100%	85.88 ± 4.33
GA002 (*C*. *theobromicola*)	0	7	9	9	92.59%	9	7	9	92.59%	7	8	7	81.48%	100%	88.88 ± 3.66

PDI (%) = x/N × 100.

PDS (%) = Σ(a + b)/N.Z × 100.

Σ(a + b) = Sum of infected fruits and their corresponding score scale.

N = Total number of sampled fruits = 3.

Z = Highest score scale = 9.

x = Number of infected fruits = 3.

### Morphological analysis

**Figure 6:**
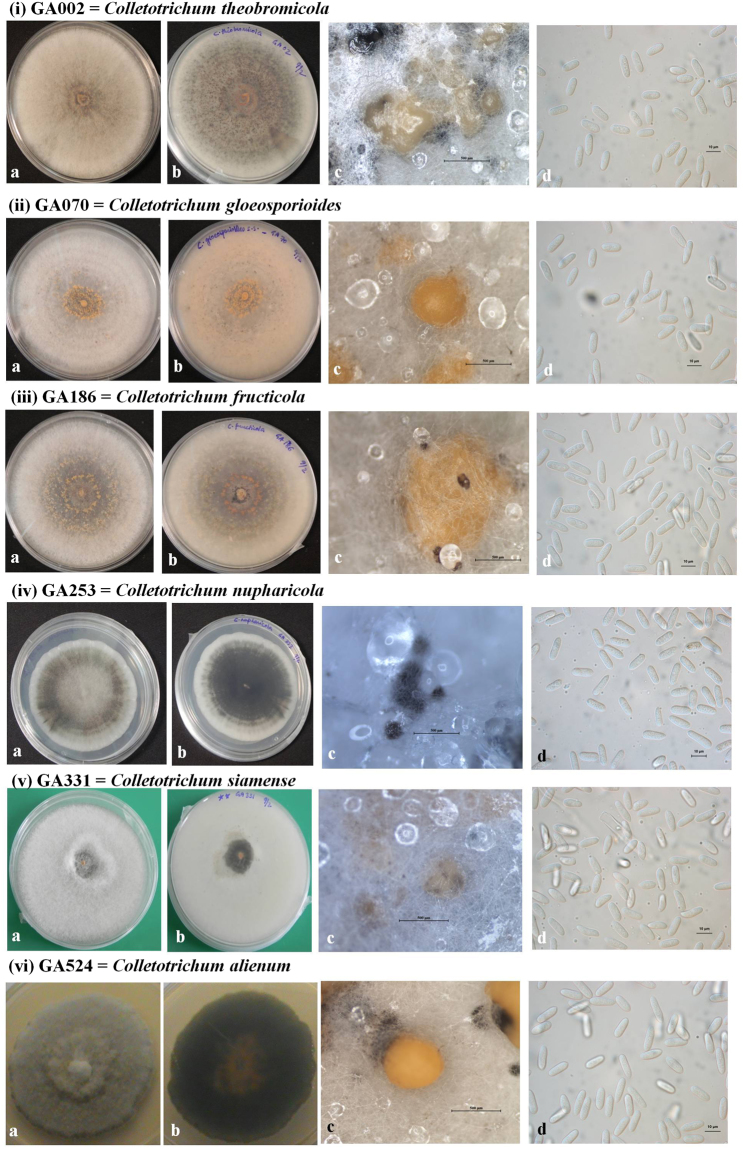
Morphological features of selected isolates of *Colletotrichum* species (**i**) *C*. *theobromicola –* GA002, (**ii**) *C*. *gloeosporioides –* GA070, (**iii**) *C*. *fructicola –* GA186, (**iv**) *C*. *nupharicola –* GA253, (**v**) *C*. *siamense –* GA331 (**vi**) *C*. *alienum –* GA524. (a) Colony morphology on PDA (front) (b) Colony morphology on PDA (reverse) (c) Conidiomata/ascomata (d) Conidia (Scale bar of c = 500 μm, Scale bar of d = 10 μm).

**Figure 7:**
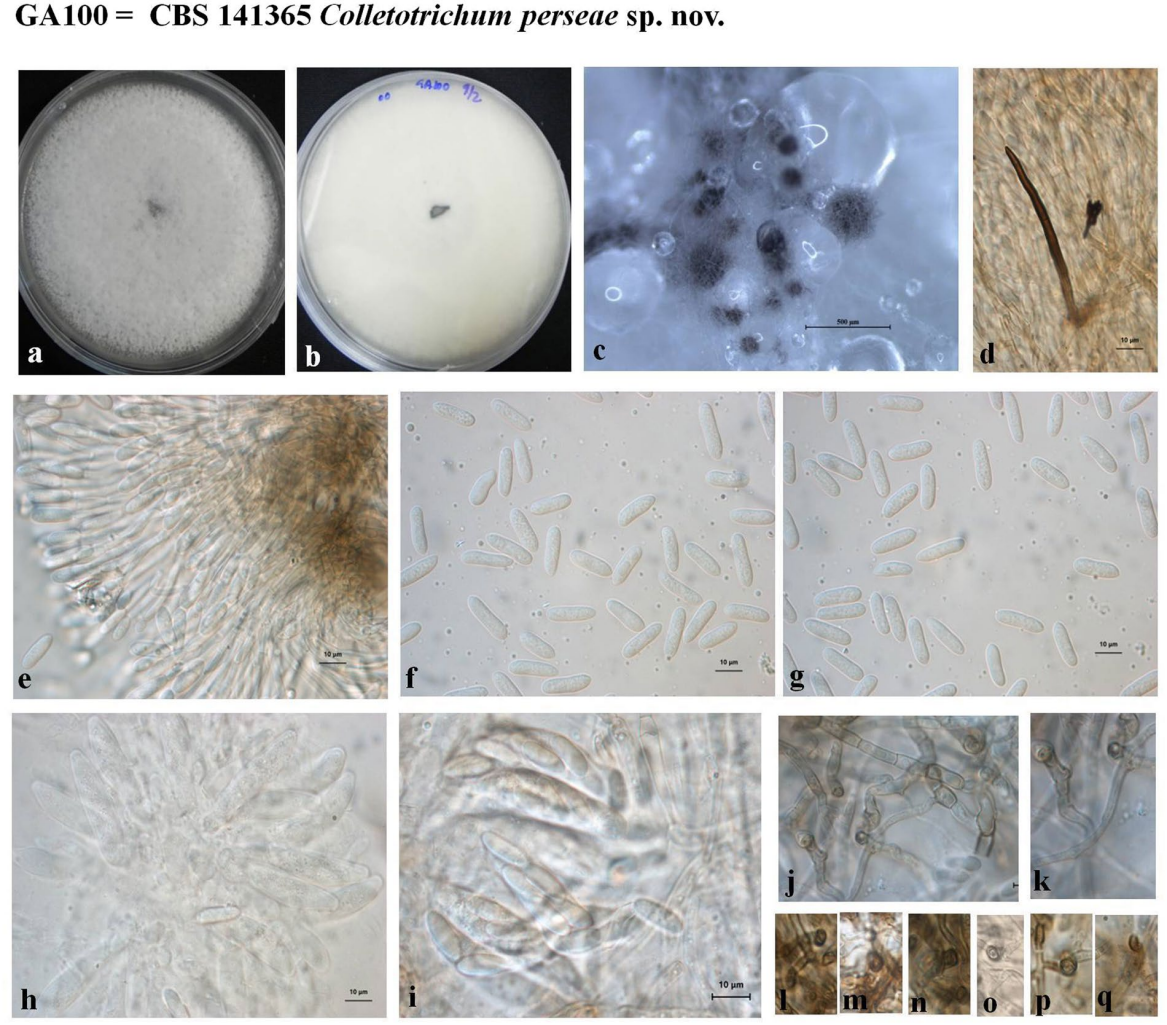
Morphological features of *Colletotrichum perseae* sp. nov. *–* GA100 = CBS 141365 (**a**) Colony morphology on PDA (front) (**b**) Colony morphology on PDA (reverse) (**c**) Ascomata (**d**) Setae (**e**) Conidiogenous cells (**f**–**g**) Conidia (**h–i**) Asci and ascospores (**j–q**) Appressoria (Scale bar of c = 500 μm, Scale bar of d-q = 10 μm).

**Figure 8:**
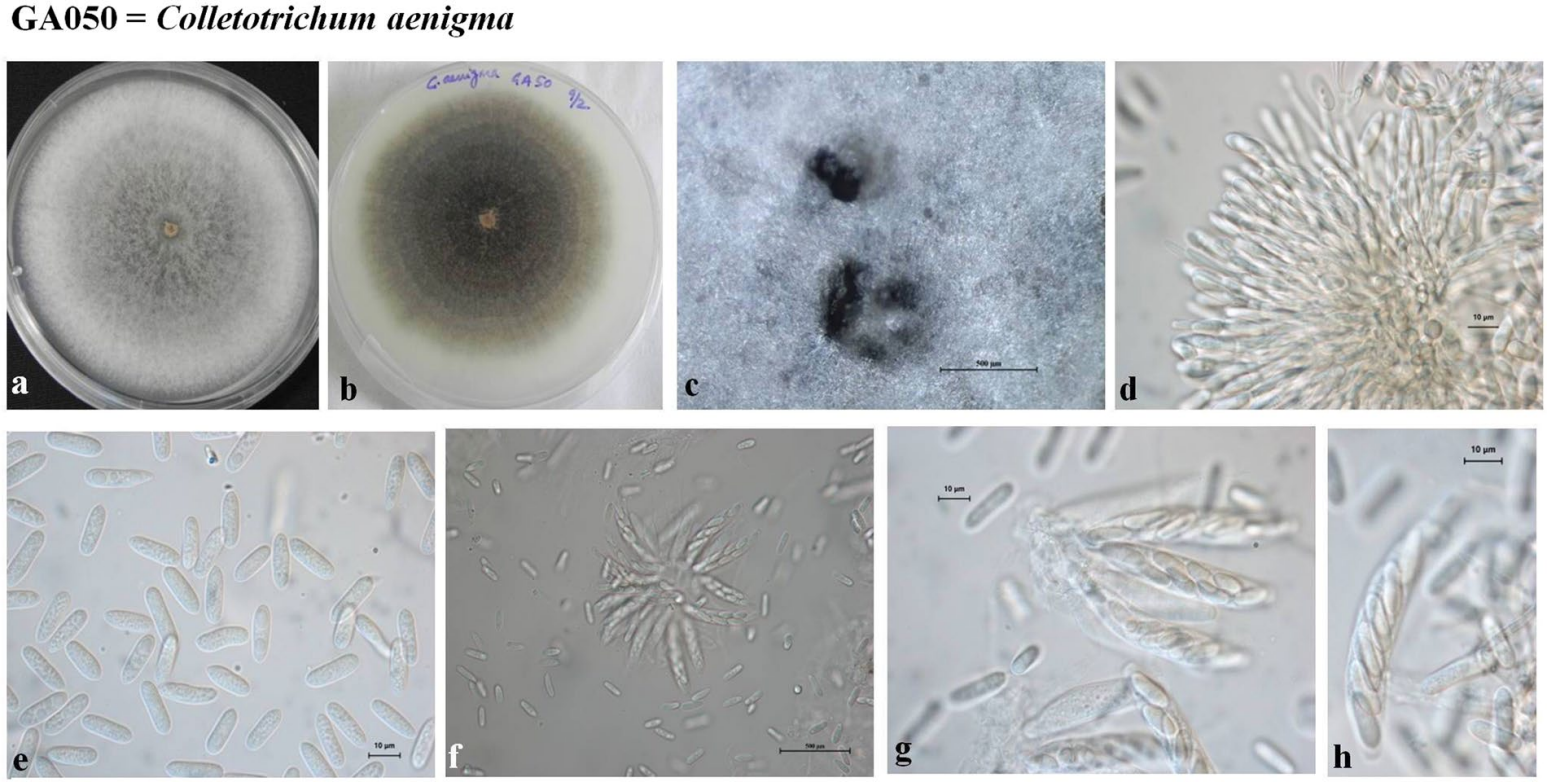
Morphological features of *Colletotrichum aenigma –* GA050 (**a**) Colony morphology on PDA (front) (**b**) Colony morphology on PDA (reverse) (**c**) Ascomata (**d**) Conidiogenous cells (**e**) Conidia (**f–h**) Asci and ascospores (Scale bar of **c**, **f** = 500 μm, Scale bar of **d**, **e**, **g**–**h** = 10 μm).

**Figure 9:**
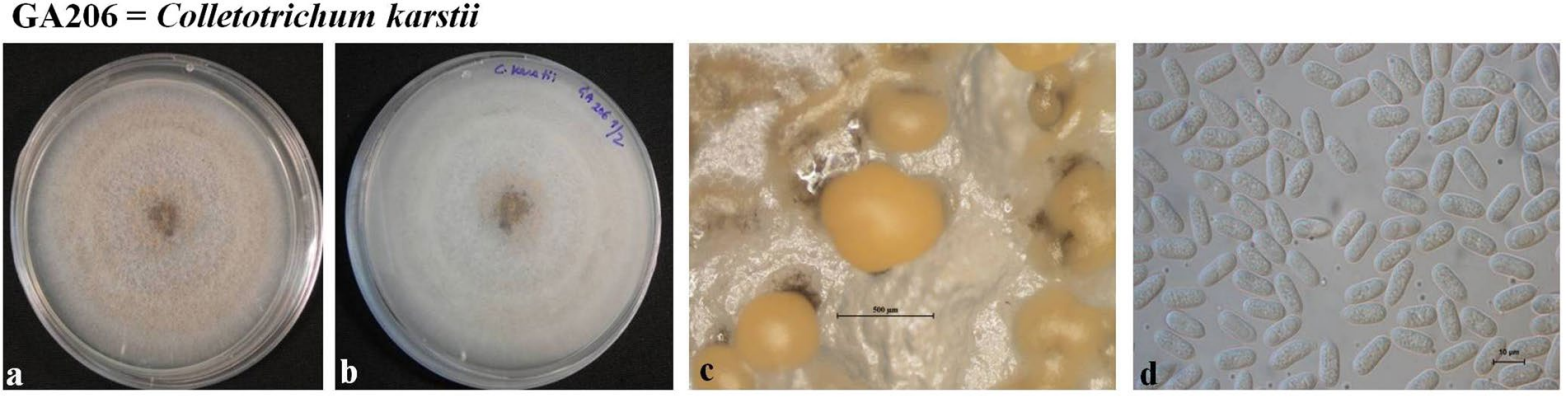
Morphological features of *Colletotrichum karstii –* GA206 (**a**) Colony morphology on PDA (front) (**b**) Colony morphology on PDA (reverse) (**c**) Conidiomata (**d**) Conidia (Scale bar of c = 500 μm, Scale bar of d = 10 μm).

**Figure 10:**
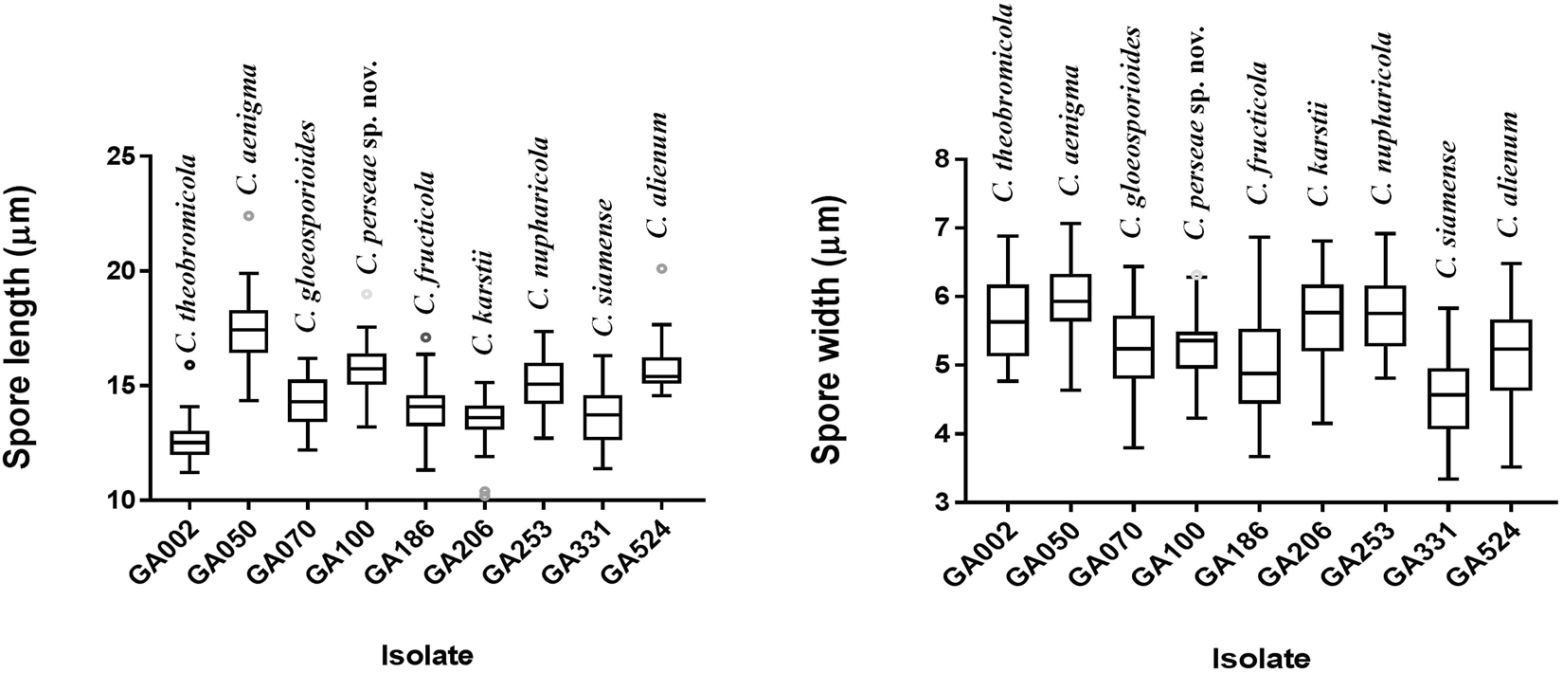
Box plots showing the variation in length and width of conidia produced by the representative isolates examined in this study.

Morphological characters (colony morphology on PDA, conidial shape and size) along with growth rates for the reference *Colletotrichum* species isolates (*C*. *aenigma* – GA050, *C*. *alienum* – GA524, *C*. *fructicola* – GA186, *C*. *gloeosporioides* – GA070, *C*. *karstii* – GA206, *C*. *nupharicola* – GA253, *C*. *perseae* sp. nov. – GA100, *C*. *siamense* – GA331, *C*. *theobromicola* – GA002) were recorded and compared to that of their respective ex-type strains (Figs 6–9, Table 4). In general, shape, size of conidia and colony growth rates were comparable to the reference strains; with minor exceptions as follows. The colony growth rate of representative isolates of *C*. *alienum* was slower, whereas that of *C*. *aenigma*, *C*. *karstii* and *C*. *nupharicola* was faster, compared to that of their respective ex-type strains. Comparisons for length and width of conidia among the different isolates were conducted according to box plots, as shown in Fig. 10.

**Table 4:** Comparison of morphological characters of selected *Colletotrichum* isolates discussed in this study with the reference type strains (*, in bold).

Taxon	Strain	Colony morphology	Conidia Length	Conidia Width	Conidia shape	Growth rate
*C*. *alienum*	GA524	cottony, grey aerial mycelium; reverse dark grey to pale orange in centre	14.5–20.0 μm Mean = 15.7 ± 0.2 μm	3.5–6.5 μm Mean = 5.0 ± 0.1 μm	cylindrical with broadly rounded ends	4.5 mm/day
***C***. ***alienum***	ICMP 12071*	cottony, grey aerial mycelium; reverse dark grey to pale orange	12.5–22.0 µm Mean = 16.5 ± 1.0 µm	3.0–6.0 µm Mean = 5.0 ± 0.50 µm	cylindrical with broadly rounded ends	8.5 mm/day
*C*. *aenigma*	GA050	aerial mycelium sparse, white to grey; reverse dark grey	14.0–22.0 μm Mean = 17.4 ± 0.2 μm	4.5–7.0 μm Mean = 5.9 ± 0.1 μm	cylindrical with broadly rounded ends	9.4 mm/day
***C***. ***aenigma***	ICMP 18608*	aerial mycelium sparse, cottony, white; reverse pale orange	12.0–16.5 µm Mean = 14.5 ± 0.5 µm	5.0–7.5 µm Mean = 6.1 ± 0.2 µm	cylindrical with broadly rounded ends	3.5 mm/day
*C*. *fructicola*	GA186	cottony, pale orange aerial mycelium; reverse pale orange	11.0–17.0 μm Mean = 14.1 ± 0.2 μm	3.5–7.0 μm Mean = 5.0 ± 0.1 μm	cylindrical with slightly tapered ends	11.6 mm/day
***C***. ***fructicola***	ICMP 18581*	cottony, dense pale grey aerial mycelium	9.7–14.0 µm Mean = 11.4 ± 0.9 µm	3.0–4.5 µm Mean = 3.5 ± 0.35 µm	cylindrical	10.7 mm/day
*C*. *gloeosporioides*	GA070	cottony, pale orange aerial mycelium; reverse dark orange	12.0–17.0 μm Mean = 14.2 ± 0.2 μm	3.5–6.5 μm Mean = 5.2 ± 0.1 μm	cylindrical, obtuse at the apex	10.5 mm/day
***C***. ***gloeosporioides***	ICMP 17821*	grey to brown, with pinkish patches; reverse dark brown	8.5–17.0 μm	3.5–4.5 μm	straight, cylindrical, obtuse at the apex	9.5 mm/day
*C*. *karstii*	GA206	yellowish to orange with whitish margins; reverse pale grey to orange	10.0–15.0 μm Mean = 13.5 ± 0.2 μm	4.0–7.0 μm Mean = 5.7 ± 0.1 μm	straight, cylindrical, rounded at both ends	8.9 mm/day
***C***. ***karstii***	CBS 127597*	grey to white aerial mycelium; reverse colorless to pale orange	14.5–17.0 μm	5.0–6.5 μm	straight, cylindrical, rounded at both ends	3.8 mm/day
*C*. *nupharicola*	GA253	grey to white velvety mycelium; reverse dark grey to white	12.5–17.5 μm Mean = 15.1 ± 0.2 μm	4.5–7.0 μm Mean = 5.7 ± 0.1 μm	fusiform to cylindrical	8.0 mm/day
***C***. ***nupharicola***	ICMP 17939*	yellowish to orange with whitish margins	14.0–53.0 μm	5.0–10.0 μm	cylindrical to clavate	4.3 mm/day
***C***. ***perseae*** **sp**. **nov**.	GA100* = CBS 141365*	cottony, white mycelium; reverse pale white to grey	13.0–19.0 μm Mean = 15.7 ± 0.1 μm	4.0–6.5 μm Mean = 5.2 ± 0.1 μm	straight, cylindrical, rounded or tapered ends	10.4 mm/day
*C*. *theobromicola*	GA002	cottony, pale orange to grey mycelium; reverse dark grey to orange	11.0–15.0 μm Mean = 12.7 ± 0.2 μm	4.5–7.0 μm Mean = 5.7 ± 0.1 μm	cylindrical, obtuse at the apex	11.1 mm/day
***C***. ***theobromicola***	ICMP 18649*	greyish mycelium	14.5–18.7 μm	4.5–5.5 μm	subcylindrical	12.5 mm/day
*C*. *siamense*	GA331	cottony, white to grey mycelium	11.5–16.5 μm Mean = 13.6 ± 0.2 μm	3.5–6.0 μm Mean = 4.6 ± 0.1 μm	fusiform to cylindrical	11.4 mm/day
***C***. ***siamense***	ICMP 18578*	Cottony, pale yellowish to pinkish mycelium	7.0–18.3 µm	3.0–6.0 µm	fusiform to cylindrical	9.1 mm/day

### Taxonomy of novel *Colletotrichum* species

*Colletotrichum perseae* sp. nov G. Sharma & S. Freeman, sp. nov. MycoBank MB819960 Fig. 7.

*Etymology*: The species epithet is derived from the host genus name *Persea americana* Mill.

*Holotype*: **Israel**, Mikve Israel, (central Israel), on *Persea americana* cv. Hass (avocado) ripe fruit rot (post-harvest), coll. S. Freeman GA100 1-12-2014, HUJIHERB-902850-FUNGI; ex-holotype culture CBS 141365.

*Asexual morph* on PDA. *Colonies* grown on PDA 105 mm diam. after 10 days, cottony, dense white aerial mycelium, with regular margin, no masses of conidial ooze, with scattered dark acervuli. In reverse, pale white with black coloration towards the center. *Vegetative hyphae* 1.5–7.5 µm diam., hyaline, smooth-walled, septate, branched. *Conidiomata* conidiophore and setae formed directly on hyphae. *Setae* approximately 50–90 µm long. *Conidiogenous* cells cylindrical, mostly 15–30 × 3.5–4 µm, arranged in closely packed palisade, conidiophores towards margins are irregularly branched, conidiogenous loci at apex. *Conidia* hyaline, straight, cylindrical, broadly rounded ends, usually tapering towards basal end, 13.0–19.0 × 4.0–6.5 µm (mean 15.7 × 5.2, n = 50). *Appressoria* irregularly shaped, dark brown, narrow.

*Sexual morph* on M3S. *Perithecia* occurring after 3–4 weeks in culture, oval or globose, scattered across plate, dark walled. *Asci* 40–65 × 8–10 µm, clavate with truncated apex, 8-spored. *Ascospores* hyaline, aseptate, slightly curved, tapering to rounded ends, 12.0–15.5 × 3.0–4.5 µm (mean 13.5 × 3.5, n = 30).

*Geographic distribution*: Known only from avocado (*Persea americana* Mill.) from Israel.

*Genetic identification*: ITS sequences are not sufficient to distinguish *C*. *perseae* from the species in the Fructicola clade. *Colletotrichum perseae* was well resolved using *ApMat* and *gs* markers.

*Other specimens examined*: **Israel**, Bet Dagan (central Israel), on *Persea americana* Mill. cv. *Ettinger* leaf spots, coll. S. Freeman GA039 12-11-2014; Mikve Israel (central Israel), on *Persea americana* cv. Hass dry leaf, coll. S. Freeman GA177 1-12-2014; Kfar Yuval (northern Israel), on *Persea americana* Mill. cv. Reed ripe fruit rot, coll. S. Freeman GA272 ( = CBS 141366) 1-4-2015; Beit Haemek (northern Israel), on *Persea americana* Mill. cv. Reed ripe fruit rot, coll. S. Freeman GA319, GA320, GA335, GA341 1-4-2015; Kfar Aza (southern Israel), on *Persea americana* Mill. cv. Hass stem rot, coll. S. Freeman GA424 1-4-2015.

***Colletotrichum aenigma*** B. Weir & P.R. Johnst., MycoBank MB563759. Fig. 8.

*Etymology*: From the Latin *aenigma*, based on the enigmatic biological and geographical distribution of this species.

*Holotype*: **Israel**, on *Persea americana* Mill., coll. S. Freeman Avo-37-4B, PDD 102233; ex-holotype culture ICMP18608.

*Asexual morph* on PDA: Detailed description provided in Weir *et al*. (2012).

*Sexual morph* on M3S: *Perithecia* occurring after 3–4 weeks in culture, scattered across plate, dark walled, globose. *Asci* 40–55 × 7–10 µm (n = 10), 8-spored. *Ascospores* slightly curved, rounded ends, 10.0–16.5 × 3.5–5.0 µm (mean 13.0 × 4.3, n = 30).

Geographic distribution: Israel (*Persea americana* Mill.) and Japan (*Pyrus pyrifolia*).

Genetic identification: ITS sequences are not sufficient to distinguish *C*. *aenigma* from *C*. *alienum* and the species in the Siamense clade. *Colletotrichum aenigma* is well resolved using *ApMat*, *gs* and *tub2* markers.

Specimens examined: **Israel**, ARO orchard, Bet Dagan (central Israel), on *Persea americana* Mill. cv. Ettinger fruit rot, coll. S. Freeman GA050, 12-11-2014; ARO orchard, Bet Dagan, on *Persea americana* cv. Ettinger green leaf spots, coll. S. Freeman GA098, 12-11-2014; Kfar Aza (southern Israel), on *Persea americana* Mill. cv. Hass fruit rot, coll. S. Freeman GA221–224, GA226–227, GA230–246, GA248–249, GA251, GA255, GA258, GA261–262, GA264–267, 01-04-2015.

## Discussion

Phenotypic plasticity within *Colletotrichum* species complexes is a key limiting factor in species delimitation. Although a polyphasic approach towards characterization of *Colletotrichum* species is a recommended strategy^18^; there is a lack of consensus among mycologists regarding the choice of markers to be used for multi-locus phylogeny^37,44^. Thus far, 11 species complexes of *Colletotrichum* have been distinguished: *C*. *acutatum*, *C*. *boninense*, *C*. *caudatum*, *C*. *dematium*, *C*. *destructivum*, *C*. *gigasporum*, *C*. *gloeosporioides*, *C*. *graminicola*, *C*. *orbiculare*, *C*. *spaethianum*, and *C*. *truncatum* ^8–12,31,39–41,45^. Besides these 11 species complexes, 23 single species and some independently evolved small clusters have been described e.g. *C*. *dracaenophilum*, *C*. *yunnanense*, *C*. *cliviae* and *C*. *araceaerum* ^8,31,46^. Each species complex is recognized by the specific epithet of a historically known or well-studied species^37^. *Colletotrichum gloeosporioides sensu lato* remains the most confusing taxa within the *Colletotrichum* genus. Following extensive taxonomic revisions^8–12,39,40^, attempts were made to investigate a potential secondary barcode for *Colletotrichum*. To date, *ApMat* and *gs* are reported to be efficient in species delimitation within the *C*. *gloeosporioides* species complex^21,22,24,26,27,42^. It is crucial to accurately identify a pathogen, for effective plant quarantine purposes, breeding programs and disease control^47,48^. This is especially important for economically important agricultural commodities such as avocado.

This study highlights the genetic heterogeneity of *Colletotrichum* populations associated with avocado anthracnose in Israel. Nine dominant *Colletotrichum* spp. causing avocado anthracnose were identified, including one new species, *C*. *perseae* sp. nov., based on multigene phylogeny, pathogenicity assays and morphology. Among the nine species identified in this paper; *C*. *aenigma*, *C*. *alienum*, *C*. *fructicola*, *C*. *gloeosporioides*, *C*. *karstii* and *C*. *siamense* have been previously reported from *Persea americana* ^11,26,43,49^. To date, only *C*. *gloeosporioides sensu lato* and *C*. *aenigma* have been reported from avocado in Israel^3,11,14,43,50^. This is the first report demonstrating pathogenicity of *C*. *persea* sp. nov., *C*. *nupharicola* and *C*. *theobromicola*, specifically in avocado. However, other *Colletotrichum* species affecting avocado in this study in Israel have been reported to attack diverse hosts. For example: *Colletotrichum aenigma* has been previously reported from *Pyrus communis*, *Citrus sinensis* (Italy) and *Pyrus pyrifolia* (Japan); *C*. *alienum* from *Malus domestica* (New Zealand); *C*. *fructicola* from *Malus domestica* (Brasil, USA), *Fragaria* sp. (Canada, USA), *Limonium* sp. (Israel), *Pyrus pyrifolia* (Japan), *Dioscorea alata* (Nigeria), *Theobroma cacao* (Panama), *Coffea arabica* (Thailand), *Mangifera indica*, *Capsicum* sp. (India); *C*. *gloeosporioides* from *Citrus* sp. (Italy, New Zealand, USA), *Mangifera indica* (South Africa, India), *Carya illinoinensis* (Australia), *Ficus* sp. (New Zealand), *Vitis vinifera*, *Pueraria lobata* (USA); *C*. *karstii* from *Annona* sp. (New Zealand, Mexico), *Anthurium* sp. (Thailand), *Eucalyptus grandis* (South Africa), *Gossypium hirsutum* (Germany), *Leucospermum* sp. (USA, Australia), *Malus* sp. (USA, Mexico, Colombia, Australia); *C*. *nupharicola* from *Nuphar lutea* (USA); *C*. *siamense* from *Hymenocallis americana* (China), *Jasminum sambac* (Vietnam), *Carica papaya* (South Africa), *Dioscorea rotundata* (Nigeria), *Malus domestica*, *Vitis vinifera*, *Fragaria* sp. (USA), *Capsicum* sp., *Mangifera indica* (India, Thailand); and *C*. *theobromicola* from *Acca sellowiana* (New Zealand), *Theobroma cacao* (Panama), *Olea europaea*, *Coffea arabica*, *Stylosanthes* sp. (Australia), *Annona diversifolia* (Mexico), *Mangifera indica* (Colombia, India), *Limonium* sp., *Cyclamen persicum* (Israel), *Fragaria* sp., and *Quercus* sp. (USA)^10,11,21,24,27,43,51–54^. This indicates the importance of cross-infection potential of different botanical hosts by more than one species of *Colletotrichum*.

In congruence with previously published studies, the *ApMat* marker proved to be superior in resolving species within the *C*. *gloeosporioides* species complex^21,22,24,26,27,42^. The *Apn2*-*Mat1-2* locus was first used for delineating populations within the *C*. *graminicola* species complex^55^. The *ApMat* gene exhibited the following advantages which established it as a promising marker for molecular systematics of the *Colletotrichum* species complexes: (a) the *apn2*-*Mat1-2* intergenic region is flanked by relatively conserved regions, useful for the design of specific primers, (b) the overall phylogenetic resolution provided by this single marker is more informative than a multigene phylogeny, and (c) the *Apmat* gene is a highly variable marker which can surpass the problems of gene tree discordance^42^. In combination with *ApMat*, *gs* was also efficient for species resolution within the *C*. *gloeosporioides* species complex, as previously observed^26^.

Pathogenic variability was also observed among the nine representative *Colletotrichum* species isolates. Pathogenicity assays confirmed that all the nine species cause anthracnose disease in avocado (Table 3). Based on percent disease severity (PDS) calculations, *C*. *aenigma* was the most virulent pathogen of avocado in Israel with 92.6 ± 7.7% values. *C*. *perseae* sp. nov. emerged as the most dominant pathogen of avocado anthracnose in Israel, occurring in all the sampled areas, totaling 354 of the 538 isolates (65.8%) included in this study. This suggests that *C*. *perseae* sp. nov. could be used as a model species for studying population structure and evolution of anthracnose in Israel. Furthermore, the occurrence of the teleomorph in *C*. *perseae* sp. nov and *C*. *aenigma* species is noteworthy, as no reports of the appearance of the sexual stage have been previously reported, albeit under artificial culture conditions; but may also appear in nature, thus contributing to the genetic diversity of these and other *Colletotrichum* species. The isolates also exhibited a pattern in geographic distribution (Supplementary Text), *C*. *theobromicola* was recovered only from samples collected from Central Israel; *C*. *persea* sp. nov. was mainly recovered from samples collected from Central and Northern Israel while *C*. *aenigma* was mainly recovered from samples collected from Southern Israel; *C*. *gloeosporioides* was mainly recovered from samples collected from Central Israel and *C*. *siamense* was mainly recovered from samples collected from the Northern and Southern Israel.

In this study percentage of isolations from different avocado tissues was significantly higher from fruits (94.9%), as compared to green/fresh leaves (19.9%), dry leaves (18%) and green/fresh twigs (10.9%), whereas only a trace percentage originated from dry and dead twigs. This is in contrast to an earlier report^56^, where large numbers of *C*. *gloeosporioides* conidia were produced on dead leaves and infected/mummified fruits in avocado trees in Australia; while minimal numbers were produced on dead twigs and none isolated from branches and green leaves. This may be associated with a dryer environment in certain areas in Australia as opposed to the constant wet, rainy winter season occurring in Israel, when avocado fruits are predominantly harvested. This elevated humidity may contribute to the relatively high percent of infected green and fresh leaves and allow survival of inoculum of the pathogen in the form of quiescent germinated apperssoria in these tissues during the dry summer season prevailing under Israeli cultivation conditions. Similarly, increased rainfall resulted in increased levels of rots in harvested avocados in certain regions of Australia^57^, and more quiescent infections became established in the temperate regions of South Africa during the rainy part of the year rather than in the dry winter months^58,59^. While spores of *C*. *gloeosporioides* were released from dead leaves entangled in the main canopy^60^, removal of this material and dead twigs from the canopy did not consistently reduce the numbers of postharvest rots in avocados while the principal means of spread to avocado fruit occurred via rain-borne inocula^61^. In another report, *C*. *gloeosporioides* conidia and perithecia were found in margins of leaf and twig lesions and in the bark from the trunks of trees^59^. The teleomorph may also occur more commonly in orchards in Israel which has remained unreported, similar to that detected *in vitro* in plates.

In summary, this work has shed light on aspects of epidemiology of avocado anthracnose in Israel, which may lay the foundation for future studies related to management of the pathogen under field conditions. The diverse genetic structure of the pathogen in Israel, further attests to the probability of the teleomorph existing under field conditions in avocado specifically, and in other economically important crops in general, affected by members of *C*. *gloeosporioides s*. *l*.

## Materials and Methods

### Sample collection

From November 2014 to April 2015, plant samples (fruit, fresh leaves and twigs, dry leaves and twigs) were collected from avocado orchards in the following locations in Israel: Kfar Yuval (Northern Israel), Beit Haemek (Northern Israel), ARO, Bet Dagan (Central Israel), Mikve Israel experimental farm (Central Israel) and Kfar Aza (Southern Israel). Initial samples were collected from plantations of Ettinger and Hass cultivars located in Mikve Israel and ARO, Bet Dagan, (Central Israel). In each orchard, five trees were selected randomly and samples of fruit, leaves (fresh green and dry leaves from the ground) and twigs (fresh green and dry twigs) were collected. During the initial isolation, only 18.6 and 3.4% of *Colletotrichum* isolates were recovered from dry leaves and twigs, along with other common fungal saprophytes such as *Aspergillus* and *Alternaria*; therefore in further samplings from Northern and Central Israel, only fruits, green leaves and green twigs were collected. In Northern Israel (from Kfar Yuval and Beit Haemek), Reed and Hass cultivars were sampled, while in Southern Israel (from Kefar Aza) Hass cultivar was sampled. From each tree five fruits, twigs and leaves of each were sampled. Samples were brought to the laboratory and maintained in a moist chamber at room temperature (20 to 25 °C) and inspected regularly for the appearance of anthracnose symptoms.

### Fungal isolation and culture conditions

Anthracnose symptoms were observed after 7–10 days in the collected fruits, leaves and twigs maintained under humid conditions. From each fruit two necrotic disease spots were selected for fungal isolation; while from each twig and leaf, five disks were removed (see below). *Colletotrichum* strains were isolated from the visible sporulation obtained on fruit lesions using the single spore method^62^. Isolation from leaves and twigs was performed initially using a tissue isolation method, whereby five sections of 1 cm^2^ size were cut from each leaf or twig near the infected area, surface sterilized with 70% ethanol for 20 seconds, 1% sodium hypochlorite (NaOCl) for 3.5 minute, washed with sterile water and dried on sterilized tissue paper. The plant tissue was then placed aseptically on Mathur’s MS semi-selective (M3S) agar medium (per liter composition: 2.5 gm of MgSO_4_.7H_2_O, 2.7 gm of KH_2_PO_4_, 1 gm of peptone, 1 gm of yeast extract, 10 gm of sucrose and 20 gm of agar), supplemented after autoclaving with iprodione at 2.5 mg/liter (Rovral 50% WP; Rhone Poulenc, France) and 0.1% lactic acid^63^. Cultures growing from twig and leaf tissue sections were further purified by the single spore method. After 5–7 days, the mycelial growth obtained was transferred onto potato dextrose agar (PDA, Difco, USA) plates, and cultured at 25 °C for morphological characterization. A total of 576 *Colletotrichum* isolates were recovered, of which morphologically identical isolates were discarded; 538 isolates were used for further characterization (Supplementary Table 1). Percentage occurrence of *Colletotrichum* isolates from fruit, leaves and twigs was calculated to determine recovery of the pathogen from the different plant parts (Fig. 1).

### DNA extraction and assessment of genetic diversity

Single spore cultures of the 538 *Colletotrichum* isolates were grown at 25 °C in liquid broth of glucose minimal media [GMM, per liter composition: 50 ml of 20 × salt solution (120 gm of NaNO_3_, 10.4 gm of KCl, 10.4 gm of MgSO_4_.7H_2_O, 30.4 gm of KH_2_PO_4_ dissolved in one liter of distilled water), 1 ml of Hunter’s trace elements solution, 10 gm of D-glucose, 5 gm of yeast extract, pH 6.5) without shaking and after 7 days fungal mycelia were harvested to isolate DNA^64^. Ap-PCR was performed using the following primers: (CAG)_5_, (GACA)_4_, (AGG)_5_ and (GACAC)_3_^14^. PCR reactions were conducted in 20 µl volume, containing 1.5 µl of total genomic DNA (50ng/µl concentration), 2 µl of 10x Taq Buffer, 1 µl of 10 µM primer, 2 µl of 25 mM MgCl_2_, 2 µl of 10 mM dNTPs, 0.2 µl of Taq Polymerase enzyme and 11.3 µl of sterile water. PCR reactions were carried out in a thermocycler (Biometra, Germany) with the following cycling parameters: initial denaturation at 95 °C for 5 minutes, followed by 29 cycles of denaturation at 95 °C for 30 seconds, annealing for 30 seconds (60 °C for CAG and AGG; 48 °C for GACAC and GACA), and extension at 72 °C for 1 minute and 30 seconds, and a final extension at 72 °C for 15 minutes. The PCR amplification and the reaction results were maintained at 4 °C until further processed. The PCR products were separated in 1.8% agarose gel (15 × 10 cm, W × L) in Tris-Acetate-EDTA buffer, at 80 V, 400 mA for 2 hours and stained with ethidium bromide (0.5 µg/ml) to visualize the banding patterns using ENDURO^TM^ GDS gel documenting system (Labnet, USA). PCR reactions were repeated three times with consistent results. Variation based on ap-PCR analysis was not quantified but diversity was interpreted according to overall banding patterns for all the 538 *Colletotrichum* isolates used in this study (Supplementary Text). Representative isolates of the different groups selected after ap-PCR were then used for sequence based analyses.

### PCR amplification and sequencing

Thirty-three representative *Colletotrichum* spp. isolates were selected according to ap-PCR for multi-locus phylogenetic analyses. PCR amplification of *act*, *cal*, *gapdh*, *tub2* and ITS regions was performed for all the isolates. In addition, *gs* and *ApMat* were amplified for isolates belonging to the *C*. *gloeosporioides* species complex, while *chs1* and *his3* gene regions were amplified for isolates belonging to the *C*. *boninense* species complex. The PCR reactions were carried out as described^11,38,42^. PCR products were purified with the Gel/PCR DNA fragments extraction kit (Geneaid, Catalogue# DF100, Taiwan), and quantified using a Nanodrop Spectrophotometer ND-1000 (Thermo, USA). Purified PCR products were sequenced by Macrogen Europe (http://www.macrogen.com) and submitted to NCBI-GenBank (Tables 1 and 2).

### Phylogenetic analyses

Phylogenetic analyses were carried out using the multigene dataset for the *C*. *gloeosporioides* species (*act*, *cal*, *gapdh*, ITS, *tub2*) and *C*. *boninense* species complexes (*act*, *cal*, *chs1*, *gapdh*, *his3*, ITS, *tub2*) using reference sequences^9,11^. In addition, analyses for *ApMat* marker, *gs* gene, 2-markers (*Apmat*, *gs*), 6-genes (*act*, *cal*, *gapdh*, *gs*, ITS, *tub2*) and 7-gene (*act*, *ApMat*, *cal*, *gapdh*, *gs*, ITS, *tub2*) were also performed for the *C*. *gloeosporioides* species complex isolates using recently published reference sequences^24,26^. Reference sequences for the newly described *Colletotrichum* species within the *C*. *gloeosporioides* species complex (*C*. *chengpingense*, *C*. *conoides*, *C*. *grossum*, *C*. *hebeinse*, *C*. *helleniense*, *C*. *henanense*, *C*. *hystricis*, *C*. *jiangxiense*, *C*. *liaoningense*, *C*. *wuxiense*) were also added to the dataset^31,65,66^. Maximum Parsimony (MP) analysis was conducted using PAUP version 4.0b10^67^. Ambiguous regions within the alignment were removed from the analyses and the gaps were considered as missing data. The trees were inferred using the heuristic search option with Tree Bisection Reconnection (TBR) branch swapping and 20 random sequence additions. Maxtrees were set to 10000, zero length branches were collapsed and all multiple parsimonious trees were saved. In addition, descriptive tree statistics, including tree length (TL), consistency index (CI), retention index (RI), rescaled consistency index (RC), and homoplasy index (HI) were recorded. The strength of clades was assessed by a bootstrap analysis with 100 replicates. The resulting trees were viewed using TreeView^68^ and edited in MEGA version 7.0.14^69,70^ and Microsoft PowerPoint version 2007. The alignment files and trees were deposited in TreeBase (www.treebase.org; Study ID: 20611). The MP trees generated in this study are shown in Figs 2–4, and Supplementary Fig. 2. Additionally, Maximum likelihood (ML) trees were generated for each dataset using “one click mode” available at the online platform for phylogenetic analysis, www.phylogeny.fr ^70^. The bootstrap support values generated using two methods (MP and ML) and the resulting tree topologies were also compared (data not shown).

### Pathogenicity assays

Pathogenicity assays were performed for representative *Colletotrichum* spp. isolates (*C*. *aenigma* – GA050, *C*. *alienum* – GA524, *C*. *fructicola* – GA186, *C*. *gloeosporioides* – GA070, *C*. *karstii* – GA206, *C*. *nupharicola* – GA253, *C*. *perseae* sp. nov. – GA100, *C*. *siamense* – GA331, *C*. *theobromicola* – GA002), essentially as described^3^. Isolates were cultured in M3S agar plates at 25 °C to induce sporulation. After 7 days, conidia were harvested by flooding the culture with 0.85% NaCl (normal saline, with 100 µl/lt Tween 80) and dislodging the conidia with a glass spreader. The conidial solution was filtered through sterile gauze to remove hyphal filaments and concentrated by centrifugation at 8000 rpm for 10 minutes at 4 °C. The supernatant was discarded and the pellet was washed and re-suspended in 1 ml of cold normal saline solution. The conidial concentration was adjusted to a final working concentration of 1 × 10^7^ conidia/ml. Disease free, fresh avocado fruits (Reed and Hass cultivars) were collected from Eyal orchard, Central Israel for pathogenicity assays. Assays were conducted in three replicates containing three fruits each, with two infection sites per fruit. The fruits were surface sterilized using 1% sodium hypochlorite solution, washed and dried using sterile filter paper. Fruits were inoculated with 7 µl of conidial suspension (1 × 10^7^ conidia/ml) at wounded (pin-pricked) and unwounded sites (3 sites per fruit). Control fruits were mock-inoculated with sterile normal saline solution. Inoculated fruits were maintained under humid conditions at 25 °C. The fruits were monitored regularly for the appearance of anthracnose symptoms. Disease severity was scored using the 0–9 point scale^65,66,71^ at 7 days post inoculation (dpi) and calculations were made as previously described^27^.

### Morphological studies

Morphological characteristics (colony color, growth rate, conidial measurements) were recorded for representative *Colletotrichum* spp. isolates (*C*. *aenigma* – GA050, *C*. *alienum* – GA524, *C*. *fructicola* – GA186, *C*. *gloeosporioides* – GA070, *C*. *karstii* – GA206, *C*. *nupharicola* – GA253, *C*. *perseae* sp. nov. – GA100, *C*. *siamense* – GA331, *C*. *theobromicola* – GA002) from 7 day old cultures grown on PDA at 25 °C. To enhance sporulation in *C*. *aenigma* and *C*. *nupharicola*, representative isolates were cultured in M3S agar medium. Ascospores, if present, were recovered from crushed ascomata. Microscopic slides were prepared in lactic acid or water. For each isolate, shape and size of conidia and conidiogenous cells were measured using differential interference contrast (DIC) microscopy (Olympus U-CMAD3, Japan). At least 30 measurements were made for the length and width of conidia and ascospores via CellB image analysis software (Olympus, Japan). Growth rate was determined by measuring colony diameter after 7 days (mm/day). Morphological characteristics are presented in Table 4.

## Electronic supplementary material

Supplementary material
