# Supplementary material for: Epidemiology, pathology and identification of Colletotrichum including a novel species associated with avocado (Persea americana) anthracnose in Israel
Source: Sci Rep. 2017 Nov 20;7:15839. doi: 10.1038/s41598-017-15946-w (PMC5696532; doi:10.1038/s41598-017-15946-w)
Supplement: Supplementary file 1 — Supplementary material [file 41598_2017_15946_MOESM1_ESM.doc]

**Epidemiology, pathology and identification of *Colletotrichum* including a novel species associated with avocado (*Persea americana*) anthracnose in Israel**

Gunjan Sharma, Marcel Maymon & Stanley Freeman^*^

Department of Plant Pathology and Weed Research, Institute of Plant Protection, Agricultural Research Organization, The Volcani Center, Rishon LeZion 7505101, Israel

^*^Corresponding author: freeman@volcani.agri.gov.il

**Supplementary Fig. 1** A representative gel picture for the representative isolates from each *Colletotrichum* species group (*C. aenigma* – GA050, *C. fructicola* – GA186, *C. gloeosporioides* – GA070, *C. karstii* – GA206, *C. nupharicola* – GA253, *C. perseae* sp. nov. – GA100, *C. siamense* – GA331, *C. theobromicola* – GA002) obtained after ap-PCR with respective primers (i) (AGG)_5_ (ii) (CAG)_5_ (iii) (GACA)_4_ (iv) (GACAC)_3_

**
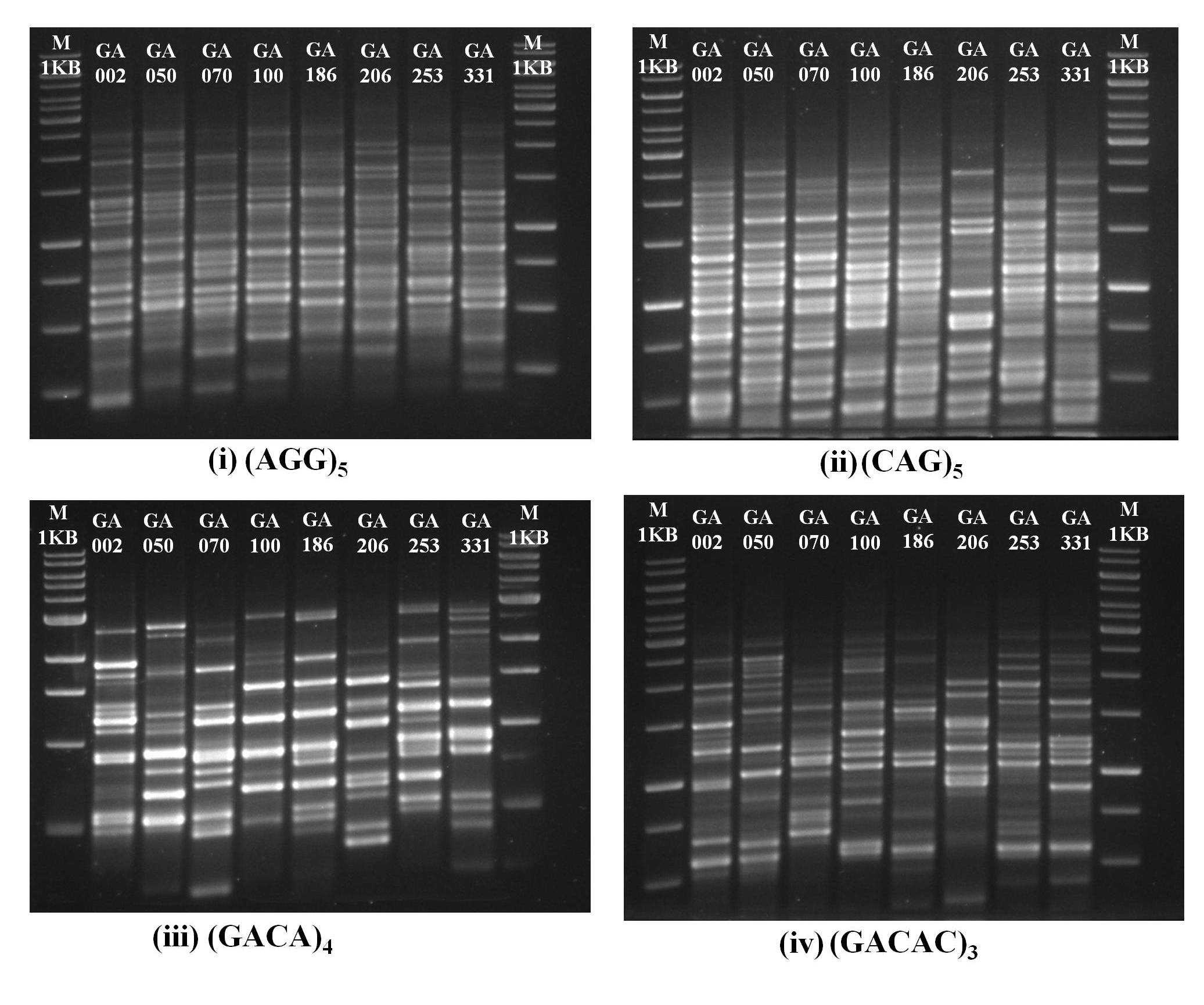
**

**Supplementary Fig. 2** One of the most parsimonious trees showing phylogenetic affinities of *Colletotrichum* isolates (highlighted in blue) belonging to the *C. gloeosporioides* species complex from Israel, obtained from heuristic search of the *ApMat*-*gs* dataset. *Colletotrichum xanthorrhoeae* ICMP 17903 is used as an outgroup, and bootstrap support values exceeding 50 %, are indicated at the nodes. (Type strains are marked with ^T^)

**
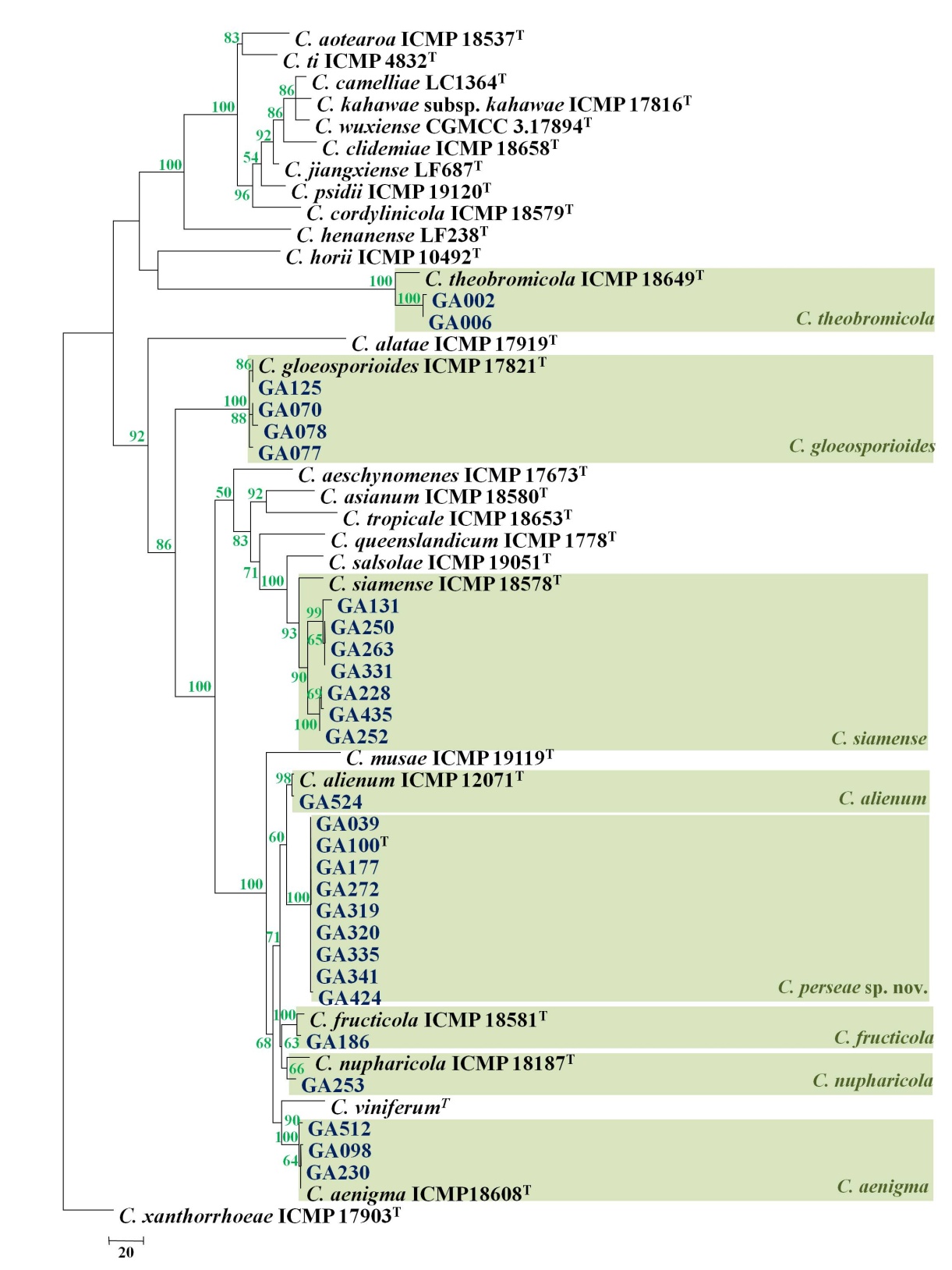
**

**Supplementary Text** Illustrative representation of each isolate within the respective group, finalized after the analysis of ap-PCR gels (Isolates in red are selected for multigene analysis)

**Group I (most common)** **(*Colletotrichum perseae*)**

001 003 004 005 007 008 009 010 011 012 013 014 015

016 017 018 019 020 021 023 024 025 026 027 028 029

030 031 032 033 034 037 038 039 040 041 042 043 044

045 048 049 051 053 054 057 058 059 060 061 062 063

064 065 067 068 069 071 072 073 076 079 080 081 082

083 084 085 088 089 090 091 092 093 094 095 096 097

099 100 101 102 103 104 105 106 107 108 109 110 111

112 113 114 115 116 117 118 119 120 122 123 124 126

127 128 129 130 133 134 135 136 137 138 139 140 141

142 143 144 145 146 147 148 149 150 151 152 153 155

156 157 158 159 160 161 162 163 164 165 166 167 169

170 171 173 174 175 176 177 178 179 180 181 182 183

184 185 187 188 189 190 191 192 193 194 195 196 197

198 199 200 201 202 203 204 207 208 210 211 212 213

214 215 216 217 218 219 220 268 270 271 272 273 274

277 282 284 287 292 298 299 305 319 320 321 322 323

324 325 326 327 328 329 332 334 335 336 337 338 339

340 341 342 343 344 345 346 347 350 351 352 353 355

356 361 363 364 365 366 367 368 369 370 371 372 373

374 375 376 377 378 379 380 381 382 383 384 385 386

387 388 389 390 391 392 393 394 395 396 397 398 399

400 401 402 403 404 405 406 407 408 409 410 411 412

414 424 436 437 438 439 440 442 443 444 445 446 447

448 449 450 451 452 453 454 455 456 457 458 459 460

469 470 471 474 475 476 477 478 481 482 483 484 486

489 490 492 493 494 495 496 497 498 499 500 501 502

503 504 505 506 507 508 509 510 511 513 515 516 517

518 525 526 527 529 530 531 532 534 535 536 537 538

**Group II (*Colletotrichum* *siamense*)**

131 132 172 225 228 229 247 250 256 259 263 269 275

276 278 279 280 281 283 285 286 288 289 290 291 293

294 295 296 297 300 301 302 303 304 306 308 309 310

310 311 312 313 314 315 316 317 318 330 331 333 348

349 354 357 358 359 362 413 416 417 418 419 420 421

422 425 426 427 428 429 430 431 432 433 434 435 441

472 473 479 487 488 491 360 461 462 463 464 465 466

467 468 480 485 514 520 521 522 523 528 533

**Group III (*Colletotrichum aenigma*)**

050 098 221 222 223 224 226 227 230 231 232 233 234

235 236 237 238 239 240 241 242 243 244 245 246 248

249 251 255 258 260 261 262 264 265 266 267 415 512

**Group IV (*Colletotrichum theobromicola*)**

002 006 022 035 036 046 047 052 055 056 066 074 075

086 087

**Group V (*Colletotricum gloeosporioides*)**

070 077 078 121 125 154 168 205 252

**Group VI (*Colletotrichum karstii*)**

206 209 423

**Group VII (*Colletotrichum fructicola*)**

186

**Group VIII (*Colletotrichum nupharicola* and *C. alienum*)**

253 254 257 307 519 524

**Color coding:** North Israel Central Israel Southern Israel

Supplementary Table 1. List of the *Colletotrichum* isolates from this study along with information on taxon, geographical location, host tissue, cultivar and date of collection (Isolates used for multi-gene sequencing mentioned in bold)

| **Isolate** | **Taxon name** | **Geographical location** | **Host tissue** | **Cultivar** | **Collected on** |
| --- | --- | --- | --- | --- | --- |
| **GA050** | *C. aenigma* | ARO Orchard, Central Israel | Fruit | Ettinger | 11/12/2016 |
| **GA098** | *C. aenigma* | ARO Orchard, Central Israel | Fresh leaf | Ettinger | 11/12/2016 |
| GA221 | *C. aenigma* | Aza Farm, Besor, South Israel | Fruit | Hass | 1/4/2015 |
| GA222 | *C. aenigma* | Aza Farm, Besor, South Israel | Fruit | Hass | 1/4/2015 |
| **GA223** | *C. aenigma* | Aza Farm, Besor, South Israel | Fruit | Hass | 1/4/2015 |
| GA224 | *C. aenigma* | Aza Farm, Besor, South Israel | Fruit | Hass | 1/4/2015 |
| GA226 | *C. aenigma* | Aza Farm, Besor, South Israel | Fruit | Hass | 1/4/2015 |
| GA227 | *C. aenigma* | Aza Farm, Besor, South Israel | Fruit | Hass | 1/4/2015 |
| **GA230** | *C. aenigma* | Aza Farm, Besor, South Israel | Fruit | Hass | 1/4/2015 |
| GA231 | *C. aenigma* | Aza Farm, Besor, South Israel | Fruit | Hass | 1/4/2015 |
| GA232 | *C. aenigma* | Aza Farm, Besor, South Israel | Fruit | Hass | 1/4/2015 |
| GA233 | *C. aenigma* | Aza Farm, Besor, South Israel | Fruit | Hass | 1/4/2015 |
| GA234 | *C. aenigma* | Aza Farm, Besor, South Israel | Fruit | Hass | 1/4/2015 |
| GA235 | *C. aenigma* | Aza Farm, Besor, South Israel | Fruit | Hass | 1/4/2015 |
| GA236 | *C. aenigma* | Aza Farm, Besor, South Israel | Fruit | Hass | 1/4/2015 |
| GA237 | *C. aenigma* | Aza Farm, Besor, South Israel | Fruit | Hass | 1/4/2015 |
| GA238 | *C. aenigma* | Aza Farm, Besor, South Israel | Fruit | Hass | 1/4/2015 |
| GA239 | *C. aenigma* | Aza Farm, Besor, South Israel | Fruit | Hass | 1/4/2015 |
| GA240 | *C. aenigma* | Aza Farm, Besor, South Israel | Fruit | Hass | 1/4/2015 |
| GA241 | *C. aenigma* | Aza Farm, Besor, South Israel | Fruit | Hass | 1/4/2015 |
| GA242 | *C. aenigma* | Aza Farm, Besor, South Israel | Fruit | Hass | 1/4/2015 |
| GA243 | *C. aenigma* | Aza Farm, Besor, South Israel | Fruit | Hass | 1/4/2015 |
| GA244 | *C. aenigma* | Aza Farm, Besor, South Israel | Fruit | Hass | 1/4/2015 |
| GA245 | *C. aenigma* | Aza Farm, Besor, South Israel | Fruit | Hass | 1/4/2015 |
| GA246 | *C. aenigma* | Aza Farm, Besor, South Israel | Fruit | Hass | 1/4/2015 |
| GA248 | *C. aenigma* | Aza Farm, Besor, South Israel | Fruit | Hass | 1/4/2015 |
| GA249 | *C. aenigma* | Aza Farm, Besor, South Israel | Fruit | Hass | 1/4/2015 |
| GA251 | *C. aenigma* | Aza Farm, Besor, South Israel | Fruit | Hass | 1/4/2015 |
| GA255 | *C. aenigma* | Aza Farm, Besor, South Israel | Fruit | Hass | 1/4/2015 |
| GA258 | *C. aenigma* | Aza Farm, Besor, South Israel | Fruit | Hass | 1/4/2015 |
| GA260 | *C. aenigma* | Aza Farm, Besor, South Israel | Fruit | Hass | 1/4/2015 |
| GA261 | *C. aenigma* | Aza Farm, Besor, South Israel | Fruit | Hass | 1/4/2015 |
| GA262 | *C. aenigma* | Aza Farm, Besor, South Israel | Fruit | Hass | 1/4/2015 |
| GA264 | *C. aenigma* | Aza Farm, Besor, South Israel | Fruit | Hass | 1/4/2015 |
| GA265 | *C. aenigma* | Aza Farm, Besor, South Israel | Fruit | Hass | 1/4/2015 |
| GA266 | *C. aenigma* | Aza Farm, Besor, South Israel | Fruit | Hass | 1/4/2015 |
| GA267 | *C. aenigma* | Aza Farm, Besor, South Israel | Fruit | Hass | 1/4/2015 |
| **GA415** | *C. aenigma* | Aza Farm, Besor, South Israel | Fresh leaf | Hass | 1/4/2015 |
| **GA512** | *C. aenigma* | Beit Haemek Orchard, North Israel | Fresh twig | Hass | 1/4/2015 |
| GA307 | *C. alienum* | Kfar Yuval Orchard, North Israel | Fruit | Reed | 1/4/2015 |
| GA519 | *C. alienum* | Kfar Yuval Orchard, North Israel | Fresh leaf | Reed | 1/4/2015 |
| **GA524** | *C. alienum* | Kfar Yuval Orchard, North Israel | Fresh twig | Reed | 1/4/2015 |
| **GA186** | *C. fructicola* | Mikve Israel Orchard, Central Israel | Dry Leaf | Hass | 1/12/2014 |
| **GA070** | *C. gloeosporioides* | ARO Orchard, Central Israel | Fruit | Ettinger | 11/12/2016 |
| **GA077** | *C. gloeosporioides* | ARO Orchard, Central Israel | Fruit | Ettinger | 11/12/2016 |
| **GA078** | *C. gloeosporioides* | ARO Orchard, Central Israel | Fruit | Ettinger | 11/12/2016 |
| GA121 | *C. gloeosporioides* | Mikve Israel Orchard, Central Israel | Fruit | Hass | 1/12/2014 |
| **GA125** | *C. gloeosporioides* | Mikve Israel Orchard, Central Israel | Fruit | Hass | 1/12/2014 |
| GA154 | *C. gloeosporioides* | Mikve Israel Orchard, Central Israel | Dry Leaf | Hass | 1/12/2014 |
| GA168 | *C. gloeosporioides* | Mikve Israel Orchard, Central Israel | Dry Twig | Hass | 1/12/2014 |
| GA205 | *C. gloeosporioides* | Mikve Israel Orchard, Central Israel | Fresh Leaf | Hass | 1/12/2014 |
| **GA206** | *C. karstii* | Mikve Israel Orchard, Central Israel | Fresh Leaf | Hass | 1/12/2014 |
| GA209 | *C. karstii* | Mikve Israel Orchard, Central Israel | Fresh Leaf | Hass | 1/12/2014 |
| **GA423** | *C. karstii* | Aza Farm, Besor, South Israel | Fresh leaf | Hass | 1/4/2015 |
| **GA253** | *C. nupharicola* | Aza Farm, Besor, South Israel | Fruit | Hass | 1/4/2015 |
| GA254 | *C. nupharicola* | Aza Farm, Besor, South Israel | Fruit | Hass | 1/4/2015 |
| GA257 | *C. nupharicola* | Aza Farm, Besor, South Israel | Fruit | Hass | 1/4/2015 |
| GA001 | *C. perseae* sp. nov. | ARO Orchard, Central Israel | Fruit | Ettinger | 11/12/2016 |
| GA003 | *C. perseae* sp. nov. | ARO Orchard, Central Israel | Fruit | Ettinger | 11/12/2016 |
| GA004 | *C. perseae* sp. nov. | ARO Orchard, Central Israel | Fruit | Ettinger | 11/12/2016 |
| GA005 | *C. perseae* sp. nov. | ARO Orchard, Central Israel | Fruit | Ettinger | 11/12/2016 |
| GA007 | *C. perseae* sp. nov. | ARO Orchard, Central Israel | Fruit | Ettinger | 11/12/2016 |
| GA008 | *C. perseae* sp. nov. | ARO Orchard, Central Israel | Fruit | Ettinger | 11/12/2016 |
| GA009 | *C. perseae* sp. nov. | ARO Orchard, Central Israel | Fruit | Ettinger | 11/12/2016 |
| GA010 | *C. perseae* sp. nov. | ARO Orchard, Central Israel | Fruit | Ettinger | 11/12/2016 |
| GA011 | *C. perseae* sp. nov. | ARO Orchard, Central Israel | Fresh leaf | Ettinger | 11/12/2016 |
| GA012 | *C. perseae* sp. nov. | ARO Orchard, Central Israel | Fresh leaf | Ettinger | 11/12/2016 |
| GA013 | *C. perseae* sp. nov. | ARO Orchard, Central Israel | Fresh leaf | Ettinger | 11/12/2016 |
| GA014 | *C. perseae* sp. nov. | ARO Orchard, Central Israel | Fresh leaf | Ettinger | 11/12/2016 |
| GA015 | *C. perseae* sp. nov. | ARO Orchard, Central Israel | Fresh leaf | Ettinger | 11/12/2016 |
| GA016 | *C. perseae* sp. nov. | ARO Orchard, Central Israel | Fresh leaf | Ettinger | 11/12/2016 |
| GA017 | *C. perseae* sp. nov. | ARO Orchard, Central Israel | Fresh leaf | Ettinger | 11/12/2016 |
| GA018 | *C. perseae* sp. nov. | ARO Orchard, Central Israel | Fresh leaf | Ettinger | 11/12/2016 |
| GA019 | *C. perseae* sp. nov. | ARO Orchard, Central Israel | Dry leaf | Ettinger | 11/12/2016 |
| GA020 | *C. perseae* sp. nov. | ARO Orchard, Central Israel | Dry leaf | Ettinger | 11/12/2016 |
| GA021 | *C. perseae* sp. nov. | ARO Orchard, Central Israel | Dry leaf | Ettinger | 11/12/2016 |
| GA023 | *C. perseae* sp. nov. | ARO Orchard, Central Israel | Dry leaf | Ettinger | 11/12/2016 |
| GA024 | *C. perseae* sp. nov. | ARO Orchard, Central Israel | Dry leaf | Ettinger | 11/12/2016 |
| GA025 | *C. perseae* sp. nov. | ARO Orchard, Central Israel | Dry leaf | Ettinger | 11/12/2016 |
| GA026 | *C. perseae* sp. nov. | ARO Orchard, Central Israel | Dry leaf | Ettinger | 11/12/2016 |
| GA027 | *C. perseae* sp. nov. | ARO Orchard, Central Israel | Fresh twig | Ettinger | 11/12/2016 |
| GA028 | *C. perseae* sp. nov. | ARO Orchard, Central Israel | Fresh twig | Ettinger | 11/12/2016 |
| GA029 | *C. perseae* sp. nov. | ARO Orchard, Central Israel | Fresh twig | Ettinger | 11/12/2016 |
| GA030 | *C. perseae* sp. nov. | ARO Orchard, Central Israel | Fresh twig | Ettinger | 11/12/2016 |
| GA031 | *C. perseae* sp. nov. | ARO Orchard, Central Israel | Fruit | Ettinger | 11/12/2016 |
| GA032 | *C. perseae* sp. nov. | ARO Orchard, Central Israel | Fruit | Ettinger | 11/12/2016 |
| GA033 | *C. perseae* sp. nov. | ARO Orchard, Central Israel | Fruit | Ettinger | 11/12/2016 |
| GA034 | *C. perseae* sp. nov. | ARO Orchard, Central Israel | Fruit | Ettinger | 11/12/2016 |
| GA037 | *C. perseae* sp. nov. | ARO Orchard, Central Israel | Fresh leaf | Ettinger | 11/12/2016 |
| GA038 | *C. perseae* sp. nov. | ARO Orchard, Central Israel | Fresh leaf | Ettinger | 11/12/2016 |
| **GA039** | *C. perseae* sp. nov. | ARO Orchard, Central Israel | Fresh leaf | Ettinger | 11/12/2016 |
| GA040 | *C. perseae* sp. nov. | ARO Orchard, Central Israel | Dry leaf | Ettinger | 11/12/2016 |
| GA041 | *C. perseae* sp. nov. | ARO Orchard, Central Israel | Dry leaf | Ettinger | 11/12/2016 |
| GA042 | *C. perseae* sp. nov. | ARO Orchard, Central Israel | Dry leaf | Ettinger | 11/12/2016 |
| GA043 | *C. perseae* sp. nov. | ARO Orchard, Central Israel | Dry leaf | Ettinger | 11/12/2016 |
| GA044 | *C. perseae* sp. nov. | ARO Orchard, Central Israel | Fresh twig | Ettinger | 11/12/2016 |
| GA045 | *C. perseae* sp. nov. | ARO Orchard, Central Israel | Fresh twig | Ettinger | 11/12/2016 |
| GA048 | *C. perseae* sp. nov. | ARO Orchard, Central Israel | Fruit | Ettinger | 11/12/2016 |
| GA049 | *C. perseae* sp. nov. | ARO Orchard, Central Israel | Fruit | Ettinger | 11/12/2016 |
| GA051 | *C. perseae* sp. nov. | ARO Orchard, Central Israel | Fruit | Ettinger | 11/12/2016 |
| GA053 | *C. perseae* sp. nov. | ARO Orchard, Central Israel | Fruit | Ettinger | 11/12/2016 |
| GA054 | *C. perseae* sp. nov. | ARO Orchard, Central Israel | Fruit | Ettinger | 11/12/2016 |
| GA057 | *C. perseae* sp. nov. | ARO Orchard, Central Israel | Fresh leaf | Ettinger | 11/12/2016 |
| GA058 | *C. perseae* sp. nov. | ARO Orchard, Central Israel | Fresh leaf | Ettinger | 11/12/2016 |
| GA059 | *C. perseae* sp. nov. | ARO Orchard, Central Israel | Fresh leaf | Ettinger | 11/12/2016 |
| GA060 | *C. perseae* sp. nov. | ARO Orchard, Central Israel | Fresh leaf | Ettinger | 11/12/2016 |
| GA061 | *C. perseae* sp. nov. | ARO Orchard, Central Israel | Fresh leaf | Ettinger | 11/12/2016 |
| GA062 | *C. perseae* sp. nov. | ARO Orchard, Central Israel | Fresh leaf | Ettinger | 11/12/2016 |
| GA063 | *C. perseae* sp. nov. | ARO Orchard, Central Israel | Dry leaf | Ettinger | 11/12/2016 |
| GA064 | *C. perseae* sp. nov. | ARO Orchard, Central Israel | Dry leaf | Ettinger | 11/12/2016 |
| GA065 | *C. perseae* sp. nov. | ARO Orchard, Central Israel | Dry leaf | Ettinger | 11/12/2016 |
| GA067 | *C. perseae* sp. nov. | ARO Orchard, Central Israel | Dry leaf | Ettinger | 11/12/2016 |
| GA068 | *C. perseae* sp. nov. | ARO Orchard, Central Israel | Dry leaf | Ettinger | 11/12/2016 |
| GA069 | *C. perseae* sp. nov. | ARO Orchard, Central Israel | Fruit | Ettinger | 11/12/2016 |
| GA071 | *C. perseae* sp. nov. | ARO Orchard, Central Israel | Fruit | Ettinger | 11/12/2016 |
| GA072 | *C. perseae* sp. nov. | ARO Orchard, Central Israel | Fruit | Ettinger | 11/12/2016 |
| GA073 | *C. perseae* sp. nov. | ARO Orchard, Central Israel | Fruit | Ettinger | 11/12/2016 |
| GA076 | *C. perseae* sp. nov. | ARO Orchard, Central Israel | Fruit | Ettinger | 11/12/2016 |
| GA079 | *C. perseae* sp. nov. | ARO Orchard, Central Israel | Fruit | Ettinger | 11/12/2016 |
| GA080 | *C. perseae* sp. nov. | ARO Orchard, Central Israel | Fruit | Ettinger | 11/12/2016 |
| GA081 | *C. perseae* sp. nov. | ARO Orchard, Central Israel | Fresh leaf | Ettinger | 11/12/2016 |
| GA082 | *C. perseae* sp. nov. | ARO Orchard, Central Israel | Dry leaf | Ettinger | 11/12/2016 |
| GA083 | *C. perseae* sp. nov. | ARO Orchard, Central Israel | Dry leaf | Ettinger | 11/12/2016 |
| GA084 | *C. perseae* sp. nov. | ARO Orchard, Central Israel | Fruit | Ettinger | 11/12/2016 |
| GA085 | *C. perseae* sp. nov. | ARO Orchard, Central Israel | Fruit | Ettinger | 11/12/2016 |
| GA088 | *C. perseae* sp. nov. | ARO Orchard, Central Israel | Fruit | Ettinger | 11/12/2016 |
| GA089 | *C. perseae* sp. nov. | ARO Orchard, Central Israel | Fruit | Ettinger | 11/12/2016 |
| GA090 | *C. perseae* sp. nov. | ARO Orchard, Central Israel | Fruit | Ettinger | 11/12/2016 |
| GA091 | *C. perseae* sp. nov. | ARO Orchard, Central Israel | Fruit | Ettinger | 11/12/2016 |
| GA092 | *C. perseae* sp. nov. | ARO Orchard, Central Israel | Fruit | Ettinger | 11/12/2016 |
| GA093 | *C. perseae* sp. nov. | ARO Orchard, Central Israel | Fruit | Ettinger | 11/12/2016 |
| GA094 | *C. perseae* sp. nov. | ARO Orchard, Central Israel | Fresh leaf | Ettinger | 11/12/2016 |
| GA095 | *C. perseae* sp. nov. | ARO Orchard, Central Israel | Fresh leaf | Ettinger | 11/12/2016 |
| GA096 | *C. perseae* sp. nov. | ARO Orchard, Central Israel | Fresh leaf | Ettinger | 11/12/2016 |
| GA097 | *C. perseae* sp. nov. | ARO Orchard, Central Israel | Fresh leaf | Ettinger | 11/12/2016 |
| GA099 | *C. perseae* sp. nov. | ARO Orchard, Central Israel | Dry twig | Ettinger | 11/12/2016 |
| **GA100** | *C. perseae* sp. nov. | Mikve Israel Orchard, Central Israel | Fruit | Hass | 1/12/2014 |
| GA101 | *C. perseae* sp. nov. | Mikve Israel Orchard, Central Israel | Fruit | Hass | 1/12/2014 |
| GA102 | *C. perseae* sp. nov. | Mikve Israel Orchard, Central Israel | Fruit | Hass | 1/12/2014 |
| GA103 | *C. perseae* sp. nov. | Mikve Israel Orchard, Central Israel | Fruit | Hass | 1/12/2014 |
| GA104 | *C. perseae* sp. nov. | Mikve Israel Orchard, Central Israel | Fruit | Hass | 1/12/2014 |
| GA105 | *C. perseae* sp. nov. | Mikve Israel Orchard, Central Israel | Fruit | Hass | 1/12/2014 |
| GA106 | *C. perseae* sp. nov. | Mikve Israel Orchard, Central Israel | Fruit | Hass | 1/12/2014 |
| GA107 | *C. perseae* sp. nov. | Mikve Israel Orchard, Central Israel | Fruit | Hass | 1/12/2014 |
| GA108 | *C. perseae* sp. nov. | Mikve Israel Orchard, Central Israel | Fruit | Hass | 1/12/2014 |
| GA109 | *C. perseae* sp. nov. | Mikve Israel Orchard, Central Israel | Fruit | Hass | 1/12/2014 |
| GA110 | *C. perseae* sp. nov. | Mikve Israel Orchard, Central Israel | Fruit | Hass | 1/12/2014 |
| GA111 | *C. perseae* sp. nov. | Mikve Israel Orchard, Central Israel | Fruit | Hass | 1/12/2014 |
| GA112 | *C. perseae* sp. nov. | Mikve Israel Orchard, Central Israel | Fruit | Hass | 1/12/2014 |
| GA113 | *C. perseae* sp. nov. | Mikve Israel Orchard, Central Israel | Fruit | Hass | 1/12/2014 |
| GA114 | *C. perseae* sp. nov. | Mikve Israel Orchard, Central Israel | Fruit | Hass | 1/12/2014 |
| GA115 | *C. perseae* sp. nov. | Mikve Israel Orchard, Central Israel | Fruit | Hass | 1/12/2014 |
| GA116 | *C. perseae* sp. nov. | Mikve Israel Orchard, Central Israel | Fruit | Hass | 1/12/2014 |
| GA117 | *C. perseae* sp. nov. | Mikve Israel Orchard, Central Israel | Fruit | Hass | 1/12/2014 |
| GA118 | *C. perseae* sp. nov. | Mikve Israel Orchard, Central Israel | Fruit | Hass | 1/12/2014 |
| GA119 | *C. perseae* sp. nov. | Mikve Israel Orchard, Central Israel | Fruit | Hass | 1/12/2014 |
| GA120 | *C. perseae* sp. nov. | Mikve Israel Orchard, Central Israel | Fruit | Hass | 1/12/2014 |
| GA122 | *C. perseae* sp. nov. | Mikve Israel Orchard, Central Israel | Fruit | Hass | 1/12/2014 |
| GA123 | *C. perseae* sp. nov. | Mikve Israel Orchard, Central Israel | Fruit | Hass | 1/12/2014 |
| GA124 | *C. perseae* sp. nov. | Mikve Israel Orchard, Central Israel | Fruit | Hass | 1/12/2014 |
| GA126 | *C. perseae* sp. nov. | Mikve Israel Orchard, Central Israel | Fruit | Hass | 1/12/2014 |
| GA127 | *C. perseae* sp. nov. | Mikve Israel Orchard, Central Israel | Fruit | Hass | 1/12/2014 |
| GA128 | *C. perseae* sp. nov. | Mikve Israel Orchard, Central Israel | Fruit | Hass | 1/12/2014 |
| GA129 | *C. perseae* sp. nov. | Mikve Israel Orchard, Central Israel | Fruit | Hass | 1/12/2014 |
| GA130 | *C. perseae* sp. nov. | Mikve Israel Orchard, Central Israel | Fruit | Hass | 1/12/2014 |
| GA133 | *C. perseae* sp. nov. | Mikve Israel Orchard, Central Israel | Fruit | Hass | 1/12/2014 |
| GA134 | *C. perseae* sp. nov. | Mikve Israel Orchard, Central Israel | Fruit | Hass | 1/12/2014 |
| GA135 | *C. perseae* sp. nov. | Mikve Israel Orchard, Central Israel | Fruit | Hass | 1/12/2014 |
| GA136 | *C. perseae* sp. nov. | Mikve Israel Orchard, Central Israel | Fruit | Hass | 1/12/2014 |
| GA137 | *C. perseae* sp. nov. | Mikve Israel Orchard, Central Israel | Fruit | Hass | 1/12/2014 |
| GA138 | *C. perseae* sp. nov. | Mikve Israel Orchard, Central Israel | Fruit | Hass | 1/12/2014 |
| GA139 | *C. perseae* sp. nov. | Mikve Israel Orchard, Central Israel | Fruit | Hass | 1/12/2014 |
| GA140 | *C. perseae* sp. nov. | Mikve Israel Orchard, Central Israel | Fruit | Hass | 1/12/2014 |
| GA141 | *C. perseae* sp. nov. | Mikve Israel Orchard, Central Israel | Fruit | Hass | 1/12/2014 |
| GA142 | *C. perseae* sp. nov. | Mikve Israel Orchard, Central Israel | Fruit | Hass | 1/12/2014 |
| GA143 | *C. perseae* sp. nov. | Mikve Israel Orchard, Central Israel | Fruit | Hass | 1/12/2014 |
| GA144 | *C. perseae* sp. nov. | Mikve Israel Orchard, Central Israel | Fruit | Hass | 1/12/2014 |
| GA145 | *C. perseae* sp. nov. | Mikve Israel Orchard, Central Israel | Fruit | Hass | 1/12/2014 |
| GA146 | *C. perseae* sp. nov. | Mikve Israel Orchard, Central Israel | Fruit | Hass | 1/12/2014 |
| GA147 | *C. perseae* sp. nov. | Mikve Israel Orchard, Central Israel | Fruit | Hass | 1/12/2014 |
| GA148 | *C. perseae* sp. nov. | Mikve Israel Orchard, Central Israel | Fruit | Hass | 1/12/2014 |
| GA149 | *C. perseae* sp. nov. | Mikve Israel Orchard, Central Israel | Fruit | Hass | 1/12/2014 |
| GA150 | *C. perseae* sp. nov. | Mikve Israel Orchard, Central Israel | Fruit | Hass | 1/12/2014 |
| GA151 | *C. perseae* sp. nov. | Mikve Israel Orchard, Central Israel | Dry Leaf | Hass | 1/12/2014 |
| GA152 | *C. perseae* sp. nov. | Mikve Israel Orchard, Central Israel | Dry Leaf | Hass | 1/12/2014 |
| GA153 | *C. perseae* sp. nov. | Mikve Israel Orchard, Central Israel | Dry Leaf | Hass | 1/12/2014 |
| GA155 | *C. perseae* sp. nov. | Mikve Israel Orchard, Central Israel | Dry Leaf | Hass | 1/12/2014 |
| GA156 | *C. perseae* sp. nov. | Mikve Israel Orchard, Central Israel | Dry Leaf | Hass | 1/12/2014 |
| GA157 | *C. perseae* sp. nov. | Mikve Israel Orchard, Central Israel | Dry Leaf | Hass | 1/12/2014 |
| GA158 | *C. perseae* sp. nov. | Mikve Israel Orchard, Central Israel | Fresh Leaf | Hass | 1/12/2014 |
| GA159 | *C. perseae* sp. nov. | Mikve Israel Orchard, Central Israel | Fresh Leaf | Hass | 1/12/2014 |
| GA160 | *C. perseae* sp. nov. | Mikve Israel Orchard, Central Israel | Fresh Leaf | Hass | 1/12/2014 |
| GA161 | *C. perseae* sp. nov. | Mikve Israel Orchard, Central Israel | Fresh Leaf | Hass | 1/12/2014 |
| GA162 | *C. perseae* sp. nov. | Mikve Israel Orchard, Central Israel | Fresh Leaf | Hass | 1/12/2014 |
| GA163 | *C. perseae* sp. nov. | Mikve Israel Orchard, Central Israel | Fresh Leaf | Hass | 1/12/2014 |
| GA164 | *C. perseae* sp. nov. | Mikve Israel Orchard, Central Israel | Fresh Leaf | Hass | 1/12/2014 |
| GA165 | *C. perseae* sp. nov. | Mikve Israel Orchard, Central Israel | Fresh Leaf | Hass | 1/12/2014 |
| GA166 | *C. perseae* sp. nov. | Mikve Israel Orchard, Central Israel | Fresh Leaf | Hass | 1/12/2014 |
| GA167 | *C. perseae* sp. nov. | Mikve Israel Orchard, Central Israel | Dry Twig | Hass | 1/12/2014 |
| GA169 | *C. perseae* sp. nov. | Mikve Israel Orchard, Central Israel | Dry Twig | Hass | 1/12/2014 |
| GA170 | *C. perseae* sp. nov. | Mikve Israel Orchard, Central Israel | Fresh Twig | Hass | 1/12/2014 |
| GA171 | *C. perseae* sp. nov. | Mikve Israel Orchard, Central Israel | Fresh Twig | Hass | 1/12/2014 |
| GA173 | *C. perseae* sp. nov. | Mikve Israel Orchard, Central Israel | Dry Leaf | Hass | 1/12/2014 |
| GA174 | *C. perseae* sp. nov. | Mikve Israel Orchard, Central Israel | Dry Leaf | Hass | 1/12/2014 |
| GA175 | *C. perseae* sp. nov. | Mikve Israel Orchard, Central Israel | Dry Leaf | Hass | 1/12/2014 |
| GA176 | *C. perseae* sp. nov. | Mikve Israel Orchard, Central Israel | Dry Leaf | Hass | 1/12/2014 |
| **GA177** | *C. perseae* sp. nov. | Mikve Israel Orchard, Central Israel | Dry Leaf | Hass | 1/12/2014 |
| GA178 | *C. perseae* sp. nov. | Mikve Israel Orchard, Central Israel | Fresh Leaf | Hass | 1/12/2014 |
| GA179 | *C. perseae* sp. nov. | Mikve Israel Orchard, Central Israel | Fresh Leaf | Hass | 1/12/2014 |
| GA180 | *C. perseae* sp. nov. | Mikve Israel Orchard, Central Israel | Fresh Leaf | Hass | 1/12/2014 |
| GA181 | *C. perseae* sp. nov. | Mikve Israel Orchard, Central Israel | Fresh Leaf | Hass | 1/12/2014 |
| GA182 | *C. perseae* sp. nov. | Mikve Israel Orchard, Central Israel | Fresh Twig | Hass | 1/12/2014 |
| GA183 | *C. perseae* sp. nov. | Mikve Israel Orchard, Central Israel | Fresh Twig | Hass | 1/12/2014 |
| GA184 | *C. perseae* sp. nov. | Mikve Israel Orchard, Central Israel | Dry Leaf | Hass | 1/12/2014 |
| GA185 | *C. perseae* sp. nov. | Mikve Israel Orchard, Central Israel | Dry Leaf | Hass | 1/12/2014 |
| GA187 | *C. perseae* sp. nov. | Mikve Israel Orchard, Central Israel | Fresh Leaf | Hass | 1/12/2014 |
| GA188 | *C. perseae* sp. nov. | Mikve Israel Orchard, Central Israel | Fresh Leaf | Hass | 1/12/2014 |
| GA189 | *C. perseae* sp. nov. | Mikve Israel Orchard, Central Israel | Fresh Leaf | Hass | 1/12/2014 |
| GA190 | *C. perseae* sp. nov. | Mikve Israel Orchard, Central Israel | Fresh Leaf | Hass | 1/12/2014 |
| GA191 | *C. perseae* sp. nov. | Mikve Israel Orchard, Central Israel | Fresh Leaf | Hass | 1/12/2014 |
| GA192 | *C. perseae* sp. nov. | Mikve Israel Orchard, Central Israel | Fresh Leaf | Hass | 1/12/2014 |
| GA193 | *C. perseae* sp. nov. | Mikve Israel Orchard, Central Israel | Fresh Leaf | Hass | 1/12/2014 |
| GA194 | *C. perseae* sp. nov. | Mikve Israel Orchard, Central Israel | Fresh Leaf | Hass | 1/12/2014 |
| GA195 | *C. perseae* sp. nov. | Mikve Israel Orchard, Central Israel | Fresh Leaf | Hass | 1/12/2014 |
| GA196 | *C. perseae* sp. nov. | Mikve Israel Orchard, Central Israel | Fresh Leaf | Hass | 1/12/2014 |
| GA197 | *C. perseae* sp. nov. | Mikve Israel Orchard, Central Israel | Fresh Leaf | Hass | 1/12/2014 |
| GA198 | *C. perseae* sp. nov. | Mikve Israel Orchard, Central Israel | Dry Leaf | Hass | 1/12/2014 |
| GA199 | *C. perseae* sp. nov. | Mikve Israel Orchard, Central Israel | Dry Leaf | Hass | 1/12/2014 |
| GA200 | *C. perseae* sp. nov. | Mikve Israel Orchard, Central Israel | Fresh Leaf | Hass | 1/12/2014 |
| GA201 | *C. perseae* sp. nov. | Mikve Israel Orchard, Central Israel | Fresh Leaf | Hass | 1/12/2014 |
| GA202 | *C. perseae* sp. nov. | Mikve Israel Orchard, Central Israel | Fresh Leaf | Hass | 1/12/2014 |
| GA203 | *C. perseae* sp. nov. | Mikve Israel Orchard, Central Israel | Fresh Leaf | Hass | 1/12/2014 |
| GA204 | *C. perseae* sp. nov. | Mikve Israel Orchard, Central Israel | Fresh Leaf | Hass | 1/12/2014 |
| GA207 | *C. perseae* sp. nov. | Mikve Israel Orchard, Central Israel | Fresh Leaf | Hass | 1/12/2014 |
| GA208 | *C. perseae* sp. nov. | Mikve Israel Orchard, Central Israel | Fresh Leaf | Hass | 1/12/2014 |
| GA210 | *C. perseae* sp. nov. | Mikve Israel Orchard, Central Israel | Fresh Leaf | Hass | 1/12/2014 |
| GA211 | *C. perseae* sp. nov. | Mikve Israel Orchard, Central Israel | Dry Twig | Hass | 1/12/2014 |
| GA212 | *C. perseae* sp. nov. | Mikve Israel Orchard, Central Israel | Dry Twig | Hass | 1/12/2014 |
| GA213 | *C. perseae* sp. nov. | Mikve Israel Orchard, Central Israel | Dry Twig | Hass | 1/12/2014 |
| GA214 | *C. perseae* sp. nov. | Mikve Israel Orchard, Central Israel | Dry Twig | Hass | 1/12/2014 |
| GA215 | *C. perseae* sp. nov. | Mikve Israel Orchard, Central Israel | Fresh Twig | Hass | 1/12/2014 |
| GA216 | *C. perseae* sp. nov. | Mikve Israel Orchard, Central Israel | Fresh Twig | Hass | 1/12/2014 |
| GA217 | *C. perseae* sp. nov. | Mikve Israel Orchard, Central Israel | Fresh Twig | Hass | 1/12/2014 |
| GA218 | *C. perseae* sp. nov. | Mikve Israel Orchard, Central Israel | Dry Leaf | Hass | 1/12/2014 |
| GA219 | *C. perseae* sp. nov. | Mikve Israel Orchard, Central Israel | Dry Leaf | Hass | 1/12/2014 |
| GA220 | *C. perseae* sp. nov. | Mikve Israel Orchard, Central Israel | Dry Leaf | Hass | 1/12/2014 |
| GA268 | *C. perseae* sp. nov. | Kfar Yuval Orchard, North Israel | Fruit | Reed | 1/4/2015 |
| GA270 | *C. perseae* sp. nov. | Kfar Yuval Orchard, North Israel | Fruit | Reed | 1/4/2015 |
| GA271 | *C. perseae* sp. nov. | Kfar Yuval Orchard, North Israel | Fruit | Reed | 1/4/2015 |
| **GA272** | *C. perseae* sp. nov. | Kfar Yuval Orchard, North Israel | Fruit | Reed | 1/4/2015 |
| GA273 | *C. perseae* sp. nov. | Kfar Yuval Orchard, North Israel | Fruit | Reed | 1/4/2015 |
| GA274 | *C. perseae* sp. nov. | Kfar Yuval Orchard, North Israel | Fruit | Reed | 1/4/2015 |
| GA277 | *C. perseae* sp. nov. | Kfar Yuval Orchard, North Israel | Fruit | Reed | 1/4/2015 |
| GA282 | *C. perseae* sp. nov. | Kfar Yuval Orchard, North Israel | Fruit | Reed | 1/4/2015 |
| GA284 | *C. perseae* sp. nov. | Kfar Yuval Orchard, North Israel | Fruit | Reed | 1/4/2015 |
| GA287 | *C. perseae* sp. nov. | Kfar Yuval Orchard, North Israel | Fruit | Reed | 1/4/2015 |
| GA292 | *C. perseae* sp. nov. | Kfar Yuval Orchard, North Israel | Fruit | Reed | 1/4/2015 |
| GA298 | *C. perseae* sp. nov. | Kfar Yuval Orchard, North Israel | Fruit | Reed | 1/4/2015 |
| GA299 | *C. perseae* sp. nov. | Kfar Yuval Orchard, North Israel | Fruit | Reed | 1/4/2015 |
| GA305 | *C. perseae* sp. nov. | Kfar Yuval Orchard, North Israel | Fruit | Reed | 1/4/2015 |
| **GA319** | *C. perseae* sp. nov. | Beit Haemek Orchard, North Israel | Fruit | Reed | 1/4/2015 |
| **GA320** | *C. perseae* sp. nov. | Beit Haemek Orchard, North Israel | Fruit | Reed | 1/4/2015 |
| GA321 | *C. perseae* sp. nov. | Beit Haemek Orchard, North Israel | Fruit | Reed | 1/4/2015 |
| GA322 | *C. perseae* sp. nov. | Beit Haemek Orchard, North Israel | Fruit | Reed | 1/4/2015 |
| GA323 | *C. perseae* sp. nov. | Beit Haemek Orchard, North Israel | Fruit | Reed | 1/4/2015 |
| GA324 | *C. perseae* sp. nov. | Beit Haemek Orchard, North Israel | Fruit | Reed | 1/4/2015 |
| GA325 | *C. perseae* sp. nov. | Beit Haemek Orchard, North Israel | Fruit | Reed | 1/4/2015 |
| GA326 | *C. perseae* sp. nov. | Beit Haemek Orchard, North Israel | Fruit | Reed | 1/4/2015 |
| GA327 | *C. perseae* sp. nov. | Beit Haemek Orchard, North Israel | Fruit | Reed | 1/4/2015 |
| GA328 | *C. perseae* sp. nov. | Beit Haemek Orchard, North Israel | Fruit | Reed | 1/4/2015 |
| GA329 | *C. perseae* sp. nov. | Beit Haemek Orchard, North Israel | Fruit | Reed | 1/4/2015 |
| GA332 | *C. perseae* sp. nov. | Beit Haemek Orchard, North Israel | Fruit | Reed | 1/4/2015 |
| GA334 | *C. perseae* sp. nov. | Beit Haemek Orchard, North Israel | Fruit | Reed | 1/4/2015 |
| **GA335** | *C. perseae* sp. nov. | Beit Haemek Orchard, North Israel | Fruit | Reed | 1/4/2015 |
| GA336 | *C. perseae* sp. nov. | Beit Haemek Orchard, North Israel | Fruit | Reed | 1/4/2015 |
| GA337 | *C. perseae* sp. nov. | Beit Haemek Orchard, North Israel | Fruit | Reed | 1/4/2015 |
| GA338 | *C. perseae* sp. nov. | Beit Haemek Orchard, North Israel | Fruit | Reed | 1/4/2015 |
| GA339 | *C. perseae* sp. nov. | Beit Haemek Orchard, North Israel | Fruit | Reed | 1/4/2015 |
| GA340 | *C. perseae* sp. nov. | Beit Haemek Orchard, North Israel | Fruit | Reed | 1/4/2015 |
| **GA341** | *C. perseae* sp. nov. | Beit Haemek Orchard, North Israel | Fruit | Reed | 1/4/2015 |
| GA342 | *C. perseae* sp. nov. | Beit Haemek Orchard, North Israel | Fruit | Reed | 1/4/2015 |
| GA343 | *C. perseae* sp. nov. | Beit Haemek Orchard, North Israel | Fruit | Reed | 1/4/2015 |
| GA344 | *C. perseae* sp. nov. | Beit Haemek Orchard, North Israel | Fruit | Reed | 1/4/2015 |
| GA345 | *C. perseae* sp. nov. | Beit Haemek Orchard, North Israel | Fruit | Reed | 1/4/2015 |
| GA346 | *C. perseae* sp. nov. | Beit Haemek Orchard, North Israel | Fruit | Reed | 1/4/2015 |
| GA347 | *C. perseae* sp. nov. | Beit Haemek Orchard, North Israel | Fruit | Reed | 1/4/2015 |
| GA350 | *C. perseae* sp. nov. | Beit Haemek Orchard, North Israel | Fruit | Reed | 1/4/2015 |
| GA351 | *C. perseae* sp. nov. | Beit Haemek Orchard, North Israel | Fruit | Reed | 1/4/2015 |
| GA352 | *C. perseae* sp. nov. | Beit Haemek Orchard, North Israel | Fruit | Reed | 1/4/2015 |
| GA353 | *C. perseae* sp. nov. | Beit Haemek Orchard, North Israel | Fruit | Reed | 1/4/2015 |
| GA355 | *C. perseae* sp. nov. | Beit Haemek Orchard, North Israel | Fruit | Reed | 1/4/2015 |
| GA356 | *C. perseae* sp. nov. | Beit Haemek Orchard, North Israel | Fruit | Reed | 1/4/2015 |
| GA361 | *C. perseae* sp. nov. | Beit Haemek Orchard, North Israel | Fruit | Reed | 1/4/2015 |
| GA363 | *C. perseae* sp. nov. | Beit Haemek Orchard, North Israel | Fruit | Reed | 1/4/2015 |
| GA364 | *C. perseae* sp. nov. | Beit Haemek Orchard, North Israel | Fruit | Reed | 1/4/2015 |
| GA365 | *C. perseae* sp. nov. | Beit Haemek Orchard, North Israel | Fruit | Reed | 1/4/2015 |
| GA366 | *C. perseae* sp. nov. | Beit Haemek Orchard, North Israel | Fruit | Hass | 1/4/2015 |
| GA367 | *C. perseae* sp. nov. | Beit Haemek Orchard, North Israel | Fruit | Hass | 1/4/2015 |
| GA368 | *C. perseae* sp. nov. | Beit Haemek Orchard, North Israel | Fruit | Hass | 1/4/2015 |
| GA369 | *C. perseae* sp. nov. | Beit Haemek Orchard, North Israel | Fruit | Hass | 1/4/2015 |
| GA370 | *C. perseae* sp. nov. | Beit Haemek Orchard, North Israel | Fruit | Hass | 1/4/2015 |
| GA371 | *C. perseae* sp. nov. | Beit Haemek Orchard, North Israel | Fruit | Hass | 1/4/2015 |
| GA372 | *C. perseae* sp. nov. | Beit Haemek Orchard, North Israel | Fruit | Hass | 1/4/2015 |
| GA373 | *C. perseae* sp. nov. | Beit Haemek Orchard, North Israel | Fruit | Hass | 1/4/2015 |
| GA374 | *C. perseae* sp. nov. | Beit Haemek Orchard, North Israel | Fruit | Hass | 1/4/2015 |
| GA375 | *C. perseae* sp. nov. | Beit Haemek Orchard, North Israel | Fruit | Hass | 1/4/2015 |
| GA376 | *C. perseae* sp. nov. | Beit Haemek Orchard, North Israel | Fruit | Hass | 1/4/2015 |
| GA377 | *C. perseae* sp. nov. | Beit Haemek Orchard, North Israel | Fruit | Hass | 1/4/2015 |
| GA378 | *C. perseae* sp. nov. | Beit Haemek Orchard, North Israel | Fruit | Hass | 1/4/2015 |
| GA379 | *C. perseae* sp. nov. | Beit Haemek Orchard, North Israel | Fruit | Hass | 1/4/2015 |
| GA380 | *C. perseae* sp. nov. | Beit Haemek Orchard, North Israel | Fruit | Hass | 1/4/2015 |
| GA381 | *C. perseae* sp. nov. | Beit Haemek Orchard, North Israel | Fruit | Hass | 1/4/2015 |
| GA382 | *C. perseae* sp. nov. | Beit Haemek Orchard, North Israel | Fruit | Hass | 1/4/2015 |
| GA383 | *C. perseae* sp. nov. | Beit Haemek Orchard, North Israel | Fruit | Hass | 1/4/2015 |
| GA384 | *C. perseae* sp. nov. | Beit Haemek Orchard, North Israel | Fruit | Hass | 1/4/2015 |
| GA385 | *C. perseae* sp. nov. | Beit Haemek Orchard, North Israel | Fruit | Hass | 1/4/2015 |
| GA386 | *C. perseae* sp. nov. | Beit Haemek Orchard, North Israel | Fruit | Hass | 1/4/2015 |
| GA387 | *C. perseae* sp. nov. | Beit Haemek Orchard, North Israel | Fruit | Hass | 1/4/2015 |
| GA388 | *C. perseae* sp. nov. | Beit Haemek Orchard, North Israel | Fruit | Hass | 1/4/2015 |
| GA389 | *C. perseae* sp. nov. | Beit Haemek Orchard, North Israel | Fruit | Hass | 1/4/2015 |
| GA390 | *C. perseae* sp. nov. | Beit Haemek Orchard, North Israel | Fruit | Hass | 1/4/2015 |
| GA391 | *C. perseae* sp. nov. | Beit Haemek Orchard, North Israel | Fruit | Hass | 1/4/2015 |
| GA392 | *C. perseae* sp. nov. | Beit Haemek Orchard, North Israel | Fruit | Hass | 1/4/2015 |
| GA393 | *C. perseae* sp. nov. | Beit Haemek Orchard, North Israel | Fruit | Hass | 1/4/2015 |
| GA394 | *C. perseae* sp. nov. | Beit Haemek Orchard, North Israel | Fruit | Hass | 1/4/2015 |
| GA395 | *C. perseae* sp. nov. | Beit Haemek Orchard, North Israel | Fruit | Hass | 1/4/2015 |
| GA396 | *C. perseae* sp. nov. | Beit Haemek Orchard, North Israel | Fruit | Hass | 1/4/2015 |
| GA397 | *C. perseae* sp. nov. | Beit Haemek Orchard, North Israel | Fruit | Hass | 1/4/2015 |
| GA398 | *C. perseae* sp. nov. | Beit Haemek Orchard, North Israel | Fruit | Hass | 1/4/2015 |
| GA399 | *C. perseae* sp. nov. | Beit Haemek Orchard, North Israel | Fruit | Hass | 1/4/2015 |
| GA400 | *C. perseae* sp. nov. | Beit Haemek Orchard, North Israel | Fruit | Hass | 1/4/2015 |
| GA401 | *C. perseae* sp. nov. | Beit Haemek Orchard, North Israel | Fruit | Hass | 1/4/2015 |
| GA402 | *C. perseae* sp. nov. | Beit Haemek Orchard, North Israel | Fruit | Hass | 1/4/2015 |
| GA403 | *C. perseae* sp. nov. | Beit Haemek Orchard, North Israel | Fruit | Hass | 1/4/2015 |
| GA404 | *C. perseae* sp. nov. | Beit Haemek Orchard, North Israel | Fruit | Hass | 1/4/2015 |
| GA405 | *C. perseae* sp. nov. | Beit Haemek Orchard, North Israel | Fruit | Hass | 1/4/2015 |
| GA406 | *C. perseae* sp. nov. | Beit Haemek Orchard, North Israel | Fruit | Hass | 1/4/2015 |
| GA407 | *C. perseae* sp. nov. | Beit Haemek Orchard, North Israel | Fruit | Hass | 1/4/2015 |
| GA408 | *C. perseae* sp. nov. | Beit Haemek Orchard, North Israel | Fruit | Hass | 1/4/2015 |
| GA409 | *C. perseae* sp. nov. | Beit Haemek Orchard, North Israel | Fruit | Hass | 1/4/2015 |
| GA410 | *C. perseae* sp. nov. | Beit Haemek Orchard, North Israel | Fruit | Hass | 1/4/2015 |
| GA411 | *C. perseae* sp. nov. | Beit Haemek Orchard, North Israel | Fruit | Hass | 1/4/2015 |
| GA412 | *C. perseae* sp. nov. | Beit Haemek Orchard, North Israel | Fruit | Hass | 1/4/2015 |
| GA414 | *C. perseae* sp. nov. | Aza Farm, Besor, South Israel | Fresh leaf | Hass | 1/4/2015 |
| **GA424** | *C. perseae* sp. nov. | Aza Farm, Besor, South Israel | Fresh twig | Hass | 1/4/2015 |
| GA436 | *C. perseae* sp. nov. | Kfar Yuval Orchard, North Israel | Fresh leaf | Reed | 1/4/2015 |
| GA437 | *C. perseae* sp. nov. | Kfar Yuval Orchard, North Israel | Fresh leaf | Reed | 1/4/2015 |
| GA438 | *C. perseae* sp. nov. | Kfar Yuval Orchard, North Israel | Fresh leaf | Reed | 1/4/2015 |
| GA439 | *C. perseae* sp. nov. | Kfar Yuval Orchard, North Israel | Fresh leaf | Reed | 1/4/2015 |
| GA440 | *C. perseae* sp. nov. | Kfar Yuval Orchard, North Israel | Fresh leaf | Reed | 1/4/2015 |
| GA442 | *C. perseae* sp. nov. | Kfar Yuval Orchard, North Israel | Fresh leaf | Reed | 1/4/2015 |
| GA443 | *C. perseae* sp. nov. | Kfar Yuval Orchard, North Israel | Fresh leaf | Reed | 1/4/2015 |
| GA444 | *C. perseae* sp. nov. | Kfar Yuval Orchard, North Israel | Fresh leaf | Reed | 1/4/2015 |
| GA445 | *C. perseae* sp. nov. | Kfar Yuval Orchard, North Israel | Fresh twig | Reed | 1/4/2015 |
| GA446 | *C. perseae* sp. nov. | Kfar Yuval Orchard, North Israel | Fresh twig | Reed | 1/4/2015 |
| GA447 | *C. perseae* sp. nov. | Kfar Yuval Orchard, North Israel | Fresh twig | Reed | 1/4/2015 |
| GA448 | *C. perseae* sp. nov. | Kfar Yuval Orchard, North Israel | Fresh twig | Reed | 1/4/2015 |
| GA449 | *C. perseae* sp. nov. | Kfar Yuval Orchard, North Israel | Fresh twig | Reed | 1/4/2015 |
| GA450 | *C. perseae* sp. nov. | Kfar Yuval Orchard, North Israel | Fresh twig | Reed | 1/4/2015 |
| GA451 | *C. perseae* sp. nov. | Kfar Yuval Orchard, North Israel | Fresh leaf | Reed | 1/4/2015 |
| GA452 | *C. perseae* sp. nov. | Kfar Yuval Orchard, North Israel | Fresh leaf | Reed | 1/4/2015 |
| GA453 | *C. perseae* sp. nov. | Kfar Yuval Orchard, North Israel | Fresh leaf | Reed | 1/4/2015 |
| GA454 | *C. perseae* sp. nov. | Kfar Yuval Orchard, North Israel | Fresh leaf | Reed | 1/4/2015 |
| GA455 | *C. perseae* sp. nov. | Kfar Yuval Orchard, North Israel | Fresh leaf | Reed | 1/4/2015 |
| GA456 | *C. perseae* sp. nov. | Kfar Yuval Orchard, North Israel | Fresh twig | Reed | 1/4/2015 |
| GA457 | *C. perseae* sp. nov. | Kfar Yuval Orchard, North Israel | Fresh twig | Reed | 1/4/2015 |
| GA458 | *C. perseae* sp. nov. | Kfar Yuval Orchard, North Israel | Fresh twig | Reed | 1/4/2015 |
| GA459 | *C. perseae* sp. nov. | Kfar Yuval Orchard, North Israel | Fresh leaf | Reed | 1/4/2015 |
| GA460 | *C. perseae* sp. nov. | Kfar Yuval Orchard, North Israel | Fresh leaf | Reed | 1/4/2015 |
| GA469 | *C. perseae* sp. nov. | Kfar Yuval Orchard, North Israel | Fresh twig | Reed | 1/4/2015 |
| GA470 | *C. perseae* sp. nov. | Kfar Yuval Orchard, North Israel | Fresh twig | Reed | 1/4/2015 |
| GA471 | *C. perseae* sp. nov. | Kfar Yuval Orchard, North Israel | Fresh twig | Reed | 1/4/2015 |
| GA474 | *C. perseae* sp. nov. | Kfar Yuval Orchard, North Israel | Fresh leaf | Reed | 1/4/2015 |
| GA475 | *C. perseae* sp. nov. | Kfar Yuval Orchard, North Israel | Fresh leaf | Reed | 1/4/2015 |
| GA476 | *C. perseae* sp. nov. | Kfar Yuval Orchard, North Israel | Fresh leaf | Reed | 1/4/2015 |
| GA477 | *C. perseae* sp. nov. | Kfar Yuval Orchard, North Israel | Fresh leaf | Reed | 1/4/2015 |
| GA478 | *C. perseae* sp. nov. | Kfar Yuval Orchard, North Israel | Fresh leaf | Reed | 1/4/2015 |
| GA481 | *C. perseae* sp. nov. | Kfar Yuval Orchard, North Israel | Fresh leaf | Reed | 1/4/2015 |
| GA482 | *C. perseae* sp. nov. | Kfar Yuval Orchard, North Israel | Fresh twig | Reed | 1/4/2015 |
| GA483 | *C. perseae* sp. nov. | Kfar Yuval Orchard, North Israel | Fresh twig | Reed | 1/4/2015 |
| GA484 | *C. perseae* sp. nov. | Kfar Yuval Orchard, North Israel | Fresh twig | Reed | 1/4/2015 |
| GA486 | *C. perseae* sp. nov. | Kfar Yuval Orchard, North Israel | Fresh twig | Reed | 1/4/2015 |
| GA489 | *C. perseae* sp. nov. | Kfar Yuval Orchard, North Israel | Fresh twig | Reed | 1/4/2015 |
| GA490 | *C. perseae* sp. nov. | Kfar Yuval Orchard, North Israel | Fresh twig | Reed | 1/4/2015 |
| GA492 | *C. perseae* sp. nov. | Beit Haemek Orchard, North Israel | Fresh twig | Reed | 1/4/2015 |
| GA493 | *C. perseae* sp. nov. | Beit Haemek Orchard, North Israel | Fresh twig | Reed | 1/4/2015 |
| GA494 | *C. perseae* sp. nov. | Beit Haemek Orchard, North Israel | Fresh leaf | Reed | 1/4/2015 |
| GA495 | *C. perseae* sp. nov. | Beit Haemek Orchard, North Israel | Fresh leaf | Reed | 1/4/2015 |
| GA496 | *C. perseae* sp. nov. | Beit Haemek Orchard, North Israel | Fresh twig | Reed | 1/4/2015 |
| GA497 | *C. perseae* sp. nov. | Beit Haemek Orchard, North Israel | Fresh twig | Reed | 1/4/2015 |
| GA498 | *C. perseae* sp. nov. | Beit Haemek Orchard, North Israel | Fresh twig | Reed | 1/4/2015 |
| GA499 | *C. perseae* sp. nov. | Beit Haemek Orchard, North Israel | Fresh twig | Reed | 1/4/2015 |
| GA500 | *C. perseae* sp. nov. | Beit Haemek Orchard, North Israel | Fresh leaf | Hass | 1/4/2015 |
| GA501 | *C. perseae* sp. nov. | Beit Haemek Orchard, North Israel | Fresh leaf | Hass | 1/4/2015 |
| GA502 | *C. perseae* sp. nov. | Beit Haemek Orchard, North Israel | Fresh leaf | Hass | 1/4/2015 |
| GA503 | *C. perseae* sp. nov. | Beit Haemek Orchard, North Israel | Fresh leaf | Hass | 1/4/2015 |
| GA504 | *C. perseae* sp. nov. | Beit Haemek Orchard, North Israel | Fresh leaf | Hass | 1/4/2015 |
| GA505 | *C. perseae* sp. nov. | Beit Haemek Orchard, North Israel | Fresh leaf | Hass | 1/4/2015 |
| GA506 | *C. perseae* sp. nov. | Beit Haemek Orchard, North Israel | Fresh leaf | Hass | 1/4/2015 |
| GA507 | *C. perseae* sp. nov. | Beit Haemek Orchard, North Israel | Fresh leaf | Hass | 1/4/2015 |
| GA508 | *C. perseae* sp. nov. | Beit Haemek Orchard, North Israel | Fresh leaf | Hass | 1/4/2015 |
| GA509 | *C. perseae* sp. nov. | Beit Haemek Orchard, North Israel | Fresh twig | Hass | 1/4/2015 |
| GA510 | *C. perseae* sp. nov. | Beit Haemek Orchard, North Israel | Fresh twig | Hass | 1/4/2015 |
| GA511 | *C. perseae* sp. nov. | Beit Haemek Orchard, North Israel | Fresh twig | Hass | 1/4/2015 |
| GA513 | *C. perseae* sp. nov. | Beit Haemek Orchard, North Israel | Fresh twig | Hass | 1/4/2015 |
| GA515 | *C. perseae* sp. nov. | Kfar Yuval Orchard, North Israel | Fresh leaf | Reed | 1/4/2015 |
| GA516 | *C. perseae* sp. nov. | Kfar Yuval Orchard, North Israel | Fresh leaf | Reed | 1/4/2015 |
| GA517 | *C. perseae* sp. nov. | Kfar Yuval Orchard, North Israel | Fresh leaf | Reed | 1/4/2015 |
| GA518 | *C. perseae* sp. nov. | Kfar Yuval Orchard, North Israel | Fresh leaf | Reed | 1/4/2015 |
| GA525 | *C. perseae* sp. nov. | Kfar Yuval Orchard, North Israel | Fresh twig | Reed | 1/4/2015 |
| GA526 | *C. perseae* sp. nov. | Kfar Yuval Orchard, North Israel | Fresh twig | Reed | 1/4/2015 |
| GA527 | *C. perseae* sp. nov. | Kfar Yuval Orchard, North Israel | Fresh twig | Reed | 1/4/2015 |
| GA529 | *C. perseae* sp. nov. | Kfar Yuval Orchard, North Israel | Fresh twig | Reed | 1/4/2015 |
| GA530 | *C. perseae* sp. nov. | Kfar Yuval Orchard, North Israel | Fresh twig | Reed | 1/4/2015 |
| GA531 | *C. perseae* sp. nov. | Beit Haemek Orchard, North Israel | Fresh leaf | Hass | 1/4/2015 |
| GA532 | *C. perseae* sp. nov. | Beit Haemek Orchard, North Israel | Fresh twig | Hass | 1/4/2015 |
| GA534 | *C. perseae* sp. nov. | Beit Haemek Orchard, North Israel | Fresh twig | Hass | 1/4/2015 |
| GA535 | *C. perseae* sp. nov. | Beit Haemek Orchard, North Israel | Fresh twig | Hass | 1/4/2015 |
| GA536 | *C. perseae* sp. nov. | Beit Haemek Orchard, North Israel | Fresh twig | Hass | 1/4/2015 |
| GA537 | *C. perseae* sp. nov. | Beit Haemek Orchard, North Israel | Fresh leaf | Hass | 1/4/2015 |
| GA538 | *C. perseae* sp. nov. | Beit Haemek Orchard, North Israel | Fresh twig | Hass | 1/4/2015 |
| **GA131** | *C. siamense* | Mikve Israel Orchard, Central Israel | Fruit | Hass | 1/12/2014 |
| GA132 | *C. siamense* | Mikve Israel Orchard, Central Israel | Fruit | Hass | 1/12/2014 |
| GA172 | *C. siamense* | Mikve Israel Orchard, Central Israel | Dry Leaf | Hass | 1/12/2014 |
| GA225 | *C. siamense* | Aza Farm, Besor, South Israel | Fruit | Hass | 1/4/2015 |
| **GA228** | *C. siamense* | Aza Farm, Besor, South Israel | Fruit | Hass | 1/4/2015 |
| GA229 | *C. siamense* | Aza Farm, Besor, South Israel | Fruit | Hass | 1/4/2015 |
| GA247 | *C. siamense* | Aza Farm, Besor, South Israel | Fruit | Hass | 1/4/2015 |
| **GA250** | *C. siamense* | Aza Farm, Besor, South Israel | Fruit | Hass | 1/4/2015 |
| **GA252** | *C. siamense* | Aza Farm, Besor, South Israel | Fruit | Hass | 1/4/2015 |
| GA256 | *C. siamense* | Aza Farm, Besor, South Israel | Fruit | Hass | 1/4/2015 |
| GA259 | *C. siamense* | Aza Farm, Besor, South Israel | Fruit | Hass | 1/4/2015 |
| **GA263** | *C. siamense* | Aza Farm, Besor, South Israel | Fruit | Hass | 1/4/2015 |
| GA269 | *C. siamense* | Kfar Yuval Orchard, North Israel | Fruit | Reed | 1/4/2015 |
| GA275 | *C. siamense* | Kfar Yuval Orchard, North Israel | Fruit | Reed | 1/4/2015 |
| GA276 | *C. siamense* | Kfar Yuval Orchard, North Israel | Fruit | Reed | 1/4/2015 |
| GA278 | *C. siamense* | Kfar Yuval Orchard, North Israel | Fruit | Reed | 1/4/2015 |
| GA279 | *C. siamense* | Kfar Yuval Orchard, North Israel | Fruit | Reed | 1/4/2015 |
| GA280 | *C. siamense* | Kfar Yuval Orchard, North Israel | Fruit | Reed | 1/4/2015 |
| GA281 | *C. siamense* | Kfar Yuval Orchard, North Israel | Fruit | Reed | 1/4/2015 |
| GA283 | *C. siamense* | Kfar Yuval Orchard, North Israel | Fruit | Reed | 1/4/2015 |
| GA285 | *C. siamense* | Kfar Yuval Orchard, North Israel | Fruit | Reed | 1/4/2015 |
| GA286 | *C. siamense* | Kfar Yuval Orchard, North Israel | Fruit | Reed | 1/4/2015 |
| GA288 | *C. siamense* | Kfar Yuval Orchard, North Israel | Fruit | Reed | 1/4/2015 |
| GA289 | *C. siamense* | Kfar Yuval Orchard, North Israel | Fruit | Reed | 1/4/2015 |
| GA290 | *C. siamense* | Kfar Yuval Orchard, North Israel | Fruit | Reed | 1/4/2015 |
| GA291 | *C. siamense* | Kfar Yuval Orchard, North Israel | Fruit | Reed | 1/4/2015 |
| GA293 | *C. siamense* | Kfar Yuval Orchard, North Israel | Fruit | Reed | 1/4/2015 |
| GA294 | *C. siamense* | Kfar Yuval Orchard, North Israel | Fruit | Reed | 1/4/2015 |
| GA295 | *C. siamense* | Kfar Yuval Orchard, North Israel | Fruit | Reed | 1/4/2015 |
| GA296 | *C. siamense* | Kfar Yuval Orchard, North Israel | Fruit | Reed | 1/4/2015 |
| GA297 | *C. siamense* | Kfar Yuval Orchard, North Israel | Fruit | Reed | 1/4/2015 |
| GA300 | *C. siamense* | Kfar Yuval Orchard, North Israel | Fruit | Reed | 1/4/2015 |
| GA301 | *C. siamense* | Kfar Yuval Orchard, North Israel | Fruit | Reed | 1/4/2015 |
| GA302 | *C. siamense* | Kfar Yuval Orchard, North Israel | Fruit | Reed | 1/4/2015 |
| GA303 | *C. siamense* | Kfar Yuval Orchard, North Israel | Fruit | Reed | 1/4/2015 |
| GA304 | *C. siamense* | Kfar Yuval Orchard, North Israel | Fruit | Reed | 1/4/2015 |
| GA306 | *C. siamense* | Kfar Yuval Orchard, North Israel | Fruit | Reed | 1/4/2015 |
| GA308 | *C. siamense* | Kfar Yuval Orchard, North Israel | Fruit | Reed | 1/4/2015 |
| GA309 | *C. siamense* | Kfar Yuval Orchard, North Israel | Fruit | Reed | 1/4/2015 |
| GA310 | *C. siamense* | Kfar Yuval Orchard, North Israel | Fruit | Reed | 1/4/2015 |
| GA311 | *C. siamense* | Kfar Yuval Orchard, North Israel | Fruit | Reed | 1/4/2015 |
| GA312 | *C. siamense* | Kfar Yuval Orchard, North Israel | Fruit | Reed | 1/4/2015 |
| GA313 | *C. siamense* | Kfar Yuval Orchard, North Israel | Fruit | Reed | 1/4/2015 |
| GA314 | *C. siamense* | Kfar Yuval Orchard, North Israel | Fruit | Reed | 1/4/2015 |
| GA315 | *C. siamense* | Kfar Yuval Orchard, North Israel | Fruit | Reed | 1/4/2015 |
| GA316 | *C. siamense* | Kfar Yuval Orchard, North Israel | Fruit | Reed | 1/4/2015 |
| GA317 | *C. siamense* | Kfar Yuval Orchard, North Israel | Fruit | Reed | 1/4/2015 |
| GA318 | *C. siamense* | Beit Haemek Orchard, North Israel | Fruit | Reed | 1/4/2015 |
| GA330 | *C. siamense* | Beit Haemek Orchard, North Israel | Fruit | Reed | 1/4/2015 |
| **GA331** | *C. siamense* | Beit Haemek Orchard, North Israel | Fruit | Reed | 1/4/2015 |
| GA333 | *C. siamense* | Beit Haemek Orchard, North Israel | Fruit | Reed | 1/4/2015 |
| GA348 | *C. siamense* | Beit Haemek Orchard, North Israel | Fruit | Reed | 1/4/2015 |
| GA349 | *C. siamense* | Beit Haemek Orchard, North Israel | Fruit | Reed | 1/4/2015 |
| GA354 | *C. siamense* | Beit Haemek Orchard, North Israel | Fruit | Reed | 1/4/2015 |
| GA357 | *C. siamense* | Beit Haemek Orchard, North Israel | Fruit | Reed | 1/4/2015 |
| GA358 | *C. siamense* | Beit Haemek Orchard, North Israel | Fruit | Reed | 1/4/2015 |
| GA359 | *C. siamense* | Beit Haemek Orchard, North Israel | Fruit | Reed | 1/4/2015 |
| GA360 | *C. siamense* | Beit Haemek Orchard, North Israel | Fruit | Reed | 1/4/2015 |
| GA362 | *C. siamense* | Beit Haemek Orchard, North Israel | Fruit | Reed | 1/4/2015 |
| GA413 | *C. siamense* | Aza Farm, Besor, South Israel | Fresh leaf | Hass | 1/4/2015 |
| GA416 | *C. siamense* | Aza Farm, Besor, South Israel | Fresh twig | Hass | 1/4/2015 |
| GA417 | *C. siamense* | Aza Farm, Besor, South Israel | Fresh leaf | Hass | 1/4/2015 |
| GA418 | *C. siamense* | Aza Farm, Besor, South Israel | Fresh leaf | Hass | 1/4/2015 |
| GA419 | *C. siamense* | Aza Farm, Besor, South Israel | Fresh twig | Hass | 1/4/2015 |
| GA420 | *C. siamense* | Aza Farm, Besor, South Israel | Fresh leaf | Hass | 1/4/2015 |
| GA421 | *C. siamense* | Aza Farm, Besor, South Israel | Fresh leaf | Hass | 1/4/2015 |
| GA422 | *C. siamense* | Aza Farm, Besor, South Israel | Fresh leaf | Hass | 1/4/2015 |
| GA425 | *C. siamense* | Aza Farm, Besor, South Israel | Fresh leaf | Hass | 1/4/2015 |
| GA426 | *C. siamense* | Aza Farm, Besor, South Israel | Fresh leaf | Hass | 1/4/2015 |
| GA427 | *C. siamense* | Aza Farm, Besor, South Israel | Fresh leaf | Hass | 1/4/2015 |
| GA428 | *C. siamense* | Aza Farm, Besor, South Israel | Fresh leaf | Hass | 1/4/2015 |
| GA429 | *C. siamense* | Aza Farm, Besor, South Israel | Fresh leaf | Hass | 1/4/2015 |
| GA430 | *C. siamense* | Aza Farm, Besor, South Israel | Fresh leaf | Hass | 1/4/2015 |
| GA431 | *C. siamense* | Aza Farm, Besor, South Israel | Fresh twig | Hass | 1/4/2015 |
| GA432 | *C. siamense* | Aza Farm, Besor, South Israel | Fresh twig | Hass | 1/4/2015 |
| GA433 | *C. siamense* | Aza Farm, Besor, South Israel | Fresh leaf | Hass | 1/4/2015 |
| GA434 | *C. siamense* | Aza Farm, Besor, South Israel | Fresh leaf | Hass | 1/4/2015 |
| **GA435** | *C. siamense* | Kfar Yuval Orchard, North Israel | Fresh leaf | Reed | 1/4/2015 |
| GA441 | *C. siamense* | Kfar Yuval Orchard, North Israel | Fresh leaf | Reed | 1/4/2015 |
| GA461 | *C. siamense* | Kfar Yuval Orchard, North Israel | Fresh leaf | Reed | 1/4/2015 |
| GA462 | *C. siamense* | Kfar Yuval Orchard, North Israel | Fresh twig | Reed | 1/4/2015 |
| GA463 | *C. siamense* | Kfar Yuval Orchard, North Israel | Fresh twig | Reed | 1/4/2015 |
| GA464 | *C. siamense* | Kfar Yuval Orchard, North Israel | Fresh twig | Reed | 1/4/2015 |
| GA465 | *C. siamense* | Kfar Yuval Orchard, North Israel | Fresh twig | Reed | 1/4/2015 |
| GA466 | *C. siamense* | Kfar Yuval Orchard, North Israel | Fresh twig | Reed | 1/4/2015 |
| GA467 | *C. siamense* | Kfar Yuval Orchard, North Israel | Fresh twig | Reed | 1/4/2015 |
| GA468 | *C. siamense* | Kfar Yuval Orchard, North Israel | Fresh twig | Reed | 1/4/2015 |
| GA472 | *C. siamense* | Kfar Yuval Orchard, North Israel | Fresh leaf | Reed | 1/4/2015 |
| GA473 | *C. siamense* | Kfar Yuval Orchard, North Israel | Fresh leaf | Reed | 1/4/2015 |
| GA479 | *C. siamense* | Kfar Yuval Orchard, North Israel | Fresh leaf | Reed | 1/4/2015 |
| GA480 | *C. siamense* | Kfar Yuval Orchard, North Israel | Fresh leaf | Reed | 1/4/2015 |
| GA485 | *C. siamense* | Kfar Yuval Orchard, North Israel | Fresh twig | Reed | 1/4/2015 |
| GA487 | *C. siamense* | Kfar Yuval Orchard, North Israel | Fresh twig | Reed | 1/4/2015 |
| GA488 | *C. siamense* | Kfar Yuval Orchard, North Israel | Fresh twig | Reed | 1/4/2015 |
| GA491 | *C. siamense* | Beit Haemek Orchard, North Israel | Fresh twig | Reed | 1/4/2015 |
| GA514 | *C. siamense* | Kfar Yuval Orchard, North Israel | Fresh leaf | Reed | 1/4/2015 |
| GA520 | *C. siamense* | Kfar Yuval Orchard, North Israel | Fresh twig | Reed | 1/4/2015 |
| GA521 | *C. siamense* | Kfar Yuval Orchard, North Israel | Fresh twig | Reed | 1/4/2015 |
| GA522 | *C. siamense* | Kfar Yuval Orchard, North Israel | Fresh twig | Reed | 1/4/2015 |
| GA523 | *C. siamense* | Kfar Yuval Orchard, North Israel | Fresh twig | Reed | 1/4/2015 |
| GA528 | *C. siamense* | Kfar Yuval Orchard, North Israel | Fresh twig | Reed | 1/4/2015 |
| GA533 | *C. siamense* | Beit Haemek Orchard, North Israel | Fresh twig | Hass | 1/4/2015 |
| **GA002** | *C. theobromicola* | ARO Orchard, Central Israel | Fruit | Ettinger | 11/12/2016 |
| **GA006** | *C. theobromicola* | ARO Orchard, Central Israel | Fruit | Ettinger | 11/12/2016 |
| GA022 | *C. theobromicola* | ARO Orchard, Central Israel | Dry leaf | Ettinger | 11/12/2016 |
| GA035 | *C. theobromicola* | ARO Orchard, Central Israel | Fruit | Ettinger | 11/12/2016 |
| GA036 | *C. theobromicola* | ARO Orchard, Central Israel | Fruit | Ettinger | 11/12/2016 |
| GA046 | *C. theobromicola* | ARO Orchard, Central Israel | Fruit | Ettinger | 11/12/2016 |
| GA047 | *C. theobromicola* | ARO Orchard, Central Israel | Fruit | Ettinger | 11/12/2016 |
| GA052 | *C. theobromicola* | ARO Orchard, Central Israel | Fruit | Ettinger | 11/12/2016 |
| GA055 | *C. theobromicola* | ARO Orchard, Central Israel | Fruit | Ettinger | 11/12/2016 |
| GA056 | *C. theobromicola* | ARO Orchard, Central Israel | Fresh leaf | Ettinger | 11/12/2016 |
| GA066 | *C. theobromicola* | ARO Orchard, Central Israel | Dry leaf | Ettinger | 11/12/2016 |
| GA074 | *C. theobromicola* | ARO Orchard, Central Israel | Fruit | Ettinger | 11/12/2016 |
| GA075 | *C. theobromicola* | ARO Orchard, Central Israel | Fruit | Ettinger | 11/12/2016 |
| GA086 | *C. theobromicola* | ARO Orchard, Central Israel | Fruit | Ettinger | 11/12/2016 |
| GA087 | *C. theobromicola* | ARO Orchard, Central Israel | Fruit | Ettinger | 11/12/2016 |

Supplementary Table 2. Comparison of morphological characters of *C. perseae* sp. nov. with the reference type strains (*) of the closely related *Colletotrichum* species

| **Taxon** | **Strain** | **Colony morphology** | **Conidia Length** | **Conidia Width** | **Conidia shape** | **Growth rate** | **Reference** |
| --- | --- | --- | --- | --- | --- | --- | --- |
| *C. perseae* sp. nov. | CBS 141365* | Cottony, dense white aerial mycelium | 13.0–19.0 µm  Mean = 15.7 ± 1.0 µm | 4.0–6.50 µm  Mean = 5.2 ± 0.47 µm | Cylindrical with broadly rounded ends | 10.5 mm/day | This study |
| *C. aenigma* | ICMP 18608* | Cottony white mycelium | 12.0–16.5 µm  Mean = 14.5 ± 0.5 µm | 5.0–7.5 µm  Mean = 6.1 ± 0.2 µm | Cylindrical with broadly rounded ends | 3.5 mm/day | Weir et al., 2012 |
| *C. alienum* | ICMP 12071* | Cottony grey aerial mycelium | 12.5–22.0 µm  Mean = 16.5 ± 1.0 µm | 3.0–6.0 µm  Mean = 5.0 ± 0.50 µm | Cylindrical with rounded ends | 8.5 mm/day | Weir et al., 2012 |
| *C. fructicola* | ICMP 18581* | Cottony, dense, pale grey aerial mycelium | 9.7–14.0 µm  Mean = 11.4 ± 0.9 µm | 3.0–4.3 µm  Mean = 3.5 ± 0.35 µm | Cylindrical | 10.7 mm/day | Prihastuti et al., 2009 |
| *C. musae* | ICMP 19119* | White to grey floccose mycelium | 11.5–19.5 µm  Mean = 14.7 ± 2.1 µm | 4.0–5.0 µm  Mean = 4.6 ± 0.41 µm | Cylindrical | 17.6 mm/day | Su et al., 2011 |
| *C. nupharicola* | ICMP 17939* | Yellowish to orange with whitish margins | 14.0–53.0 µm | 5.0–10.0 µm | Cylindrical to clavate | 4.2 mm/day | Johnsson et al., 1997 |
| *C. viniferum* | CBS 130643* | White to grey mycelium | 12.0–16.0 µm  Mean = 13.8 ± 0.9 µm | 4.5–6.0 µm  Mean = 5.4 ± 0.38 µm | Cylindrical with obtuse to slightly rounded ends | 8.2 mm/day | Peng et al., 2013 |
